# Germline de novo mutation rate of the highly heterozygous amphioxus genome

**DOI:** 10.1093/molbev/msag017

**Published:** 2026-01-14

**Authors:** Jing Xue, Lei Tao, Junwei Cao, Liang Wu, Guang Li, Cai Li

**Affiliations:** State Key Laboratory of Biocontrol, School of Life Sciences, Guangdong Provincial Key Laboratory for Aquatic Economic Animals, Sun Yat-sen University, Guangzhou, Guangdong, China; Chinese Academy of Sciences, Guangzhou Institutes of Biomedicine and Health, Guangzhou, Guangdong, China; MOE Key Laboratory of Freshwater Fish Reproduction and Development, School of Life Sciences, Southwest University, Chongqing, China; State Key Laboratory of Biocontrol, School of Life Sciences, Guangdong Provincial Key Laboratory for Aquatic Economic Animals, Sun Yat-sen University, Guangzhou, Guangdong, China; School of Life Sciences, Xiamen University, Xiamen, Fujian, China; State Key Laboratory of Biocontrol, School of Life Sciences, Guangdong Provincial Key Laboratory for Aquatic Economic Animals, Sun Yat-sen University, Guangzhou, Guangdong, China; School of Life Sciences, Xiamen University, Xiamen, Fujian, China; State Key Laboratory of Biocontrol, School of Life Sciences, Guangdong Provincial Key Laboratory for Aquatic Economic Animals, Sun Yat-sen University, Guangzhou, Guangdong, China

## Abstract

Germline de novo mutations (DNMs) are the ultimate source of heritable variation, yet their patterns in highly heterozygous genomes remain poorly understood. Amphioxus, an early-branching chordate with exceptionally high genomic heterozygosity (3.2% to 4.2% in sequenced species), offers a unique model to explore mutational dynamics in such contexts. It is unclear whether high heterozygosity in amphioxus is due to a large effective population size, an increased mutation rate, or both. Here, we perform deep short-read whole-genome sequencing of a two-generation pedigree of the amphioxus *Branchiostoma floridae* comprising two parents and 104 offspring and develop a framework based on allele-aware parental assemblies as the reference to accurately identify DNMs. We detect 242 high-confidence DNMs, yielding a genome-wide mutation rate of 5.89 × 10^−9^ per base per generation, which is comparable to that of vertebrates. Combining this estimate with observed nucleotide diversity, we obtain an effective population size of ∼1.7 million, indicating that the elevated heterozygosity mainly results from a large effective population size. We observe no sex bias when considering all DNMs but a paternal-origin bias for early-occurring ones. Amphioxus harbors a much smaller fraction of CpG>TpG DNMs relative to vertebrates, attributable to its low methylation levels. We also investigate putative postzygotic mutations in the offspring, revealing an unexpected paternal-origin bias. These suggest some distinct mutational mechanisms in amphioxus. Our study not only provides the first DNM measurement for amphioxus but also offers a generalizable strategy for studying DNMs in highly heterozygous genomes, facilitating mutation rate studies across chordates and other lineages.

## Introduction

Germline de novo mutations (DNMs) serve as the ultimate source of genetic variation and are critical for biological evolution. These mutations arise as a consequence of replication errors or mis-repaired/un-repaired DNA damage. The DNM rate, the frequency at which DNMs occur, is an important parameter for many genetic and evolutionary analyses. Because most mutations tend to be deleterious, organisms incur considerable adaptive costs to minimize such errors and damage, thereby maintaining mutation rates at an extremely low level, ranging from 10^−11^ to 10^−7^ per base per generation in eukaryotes (Lynch et al. 2023; Wang and Obbard 2023). Thanks to the advances in sequencing and computational methods, in the past decade, direct detection of DNMs by sequencing parent-offspring trios has been available for many species, particularly for vertebrates (Besenbacher et al. 2019; Bergeron et al. 2021; Wang et al. 2022, 2025; Zhang et al. 2022, 2023, 2025; Bergeron et al. 2023; Lin et al. 2023; Sendell-Price et al. 2023; Liang et al. 2024; Porubsky et al. 2025; Prentout et al. 2025; Versoza et al. 2025), providing insight into inter-species mutation rate variation and underlying causes.

Mutation rates also vary across genomic positions, developmental stages, parental origins, and mutation types. For example, in mammals the C>T mutation rate at CpG sites is about one order of magnitude higher than the genome-wide average, which is mainly caused by spontaneous deamination of methylated cytosines (Hodgkinson and Eyre-Walker 2011). A recent study in amniotes found that while both sexes exhibit similar mutation rates early in development, the male germline accumulates more mutations after sexual differentiation (de Manuel et al. 2022), highlighting the influence of gametogenesis on mutation accumulation. Despite the progress, many details of underlying mutational mechanisms remain uncovered.

Amphioxus (cephalochordates) represents the earliest-branching lineage of extant chordates, diverging from the common ancestor of vertebrates and tunicates over 500 million years ago, serving as a key model for understanding vertebrate origins (Holland and Holland 2017). Although amphioxus shares a basic body plan with vertebrates, it is anatomically simpler, and its genome has undergone fewer lineage-specific rearrangements compared to tunicates (Delsuc et al. 2006; Putnam et al. 2008). Notably, amphioxus displays an exceptionally high level of genomic heterozygosity (3.2% to 4.2% in sequenced species) (Huang et al. 2023), among the highest observed in chordates (Bi et al. 2020). This level of heterozygosity is nearly 40-fold higher than the average ∼0.1% observed in humans and 10- to 20-fold higher than the 0.14% to 0.29% typically seen in wild house mice (Agwamba and Nachman 2023). Under neutral evolution, the equilibrium level of heterozygosity is proportional to both mutation rate and effective population size (Kimura 1983). In the absence of accurate mutation rate measurement, it is unclear whether the high heterozygosity in amphioxus is due to a large effective population size, an increased mutation rate, or both.

High heterozygosity is not unique to amphioxus—many species across diverse clades, such as marine invertebrates (Small et al. 2007; Ketchum et al. 2020; Nong et al. 2020; Shingate et al. 2020; Penaloza et al. 2021; Wooldridge et al. 2025), insects (Mackintosh et al. 2019; Kim et al. 2021; Bastide et al. 2022; Garcia-Berro et al. 2023; Deng et al. 2024; Jiang et al. 2025), and plants (Bredeson et al. 2016; Tang et al. 2016; Shen et al. 2018; Wang et al. 2020; Xian et al. 2025), also exhibit elevated genomic diversity. Interestingly, multiple sequenced tunicate species such as sea squirts (Small et al. 2007; Wei et al. 2020) and larvaceans (Denoeud et al. 2010; Bliznina et al. 2021), which are close relatives of vertebrates, also possess genomes with high heterozygosity. Yet, for these species, direct estimates of the germline mutation rate remain unavailable, largely due to technical challenges in working with highly heterozygous genomes (Garimella et al. 2020). The high heterozygosity presents significant challenges to key genomic analyses such as genome assembly (Kajitani et al. 2014; Zhang et al. 2020), read alignment (Landan and Graur 2009), and variant detection (Gan et al. 2011; Veeckman et al. 2019). Indeed, previous research in macaques has shown that even gold-standard pipelines for DNM detection exhibit elevated error rates in highly heterozygous genomes (Bergeron et al. 2022). As a result, the evolutionary forces shaping high genomic diversity in these lineages remain poorly understood.

Amphioxus, with its exceptionally high heterozygosity and relatively small genomes (∼500 Mb) (Huang et al. 2023), represents an ideal model system for addressing these questions. Direct measurement of DNMs in the amphioxus genome is not only critical for estimating its mutation rate and studying mutational mechanisms, but also highly informative for a broader set of species with extreme genomic diversity.

In this study, we generated deep short-read sequencing data for a two-generation pedigree (two parents and 104 F1 offspring) of *Branchiostoma floridae*, to systematically study DNMs in the amphioxus. To overcome the challenges in alignment and variant detection, we developed a workflow leveraging the high heterozygosity to assemble the contig-level haplotypes of parental genomes, thereby allowing mapping offspring reads to the parental genomes for identifying DNMs. This new and cost-effective strategy can be generalized to other species with high heterozygosity. With the detected DNMs and estimated mutation rate (5.89^−10^ per base per generation), we concluded that the high heterozygosity in amphioxus is in fact caused by the large effective population size. We further investigated multiple aspects of DNMs as well as postzygotic mutations (PZMs) in amphioxus, providing insights into mutational mechanisms in this basal chordate lineage.

## Results

### An analysis strategy based on parental genome assembly for detecting DNMs

To identify DNMs, we performed deep short-read whole-genome sequencing (WGS) for two parental individuals and 104 F1 offspring of a *B. floridae* family, yielding an average coverage of 83× per sample (ranging from 49× to 141×; 85× for maternal and 75× for paternal) (Figure S1 and Table S1).

K-mer analysis revealed that the heterozygosity of the parental genomes was approximately 3% (Figure S2). The high level of heterozygosity in the *B. floridae* genome poses a formidable challenge for conventional DNM detection pipelines based on aligning reads of parents and offspring against a public reference genome (Bergeron et al. 2022). Because of low sequence similarity between our sequencing data and the public reference genome (Huang et al. 2023), many reads in our sequencing data cannot be correctly mapped to the reference genome by common short-read aligners (e.g. BWA), leading to unreliable variant calling results. Although the mapping rate of parental reads to the public reference genome was 96% (Figure S1), ∼10% of all sequenced bases were extensively soft-clipped (Figure S3) and thus effectively invisible to conventional variant callers. Using these alignments for variant calling by GATK yielded only ∼2 million heterozygous variants in each parent, corresponding to ∼0.6% heterozygosity, far below the ∼3% heterozygosity estimated from *k*-mer frequency analysis.

By simulating sequencing reads with ART (Huang et al. 2012), we estimated that, when the sequence difference between the query genome and the reference genome is 4%, >2% of reads are mapped to wrong genomic regions (Table S2) and such problematic alignments are difficult to distinguish in real datasets when calling variants. For amphioxus, and for other species with similarly extreme levels of genomic heterozygosity, these considerations indicate that relying solely on a haploid reference genome makes it extremely difficult to obtain a robust and comprehensive set of DNMs.

**Figure 1:**
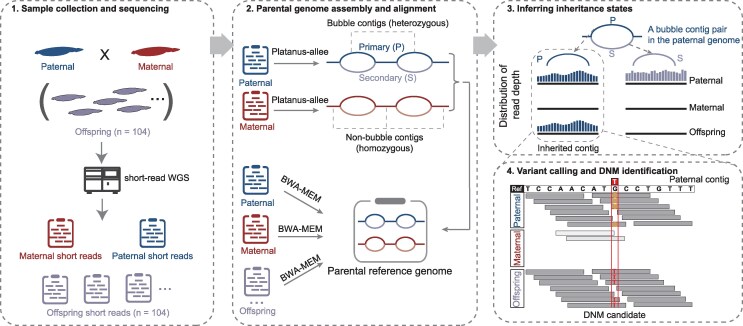
Schematic of the workflow for detecting DNMs. We collected DNA samples from a *B. floridae* family of two parents and 104 F1 offspring and performed deep short-read sequencing (∼50× or higher) for all individuals. Using the parental WGS data, we assembled allele-aware parental genomes with Platanus-allee (Kajitani et al. 2019) and combined the resulting assemblies to form a parental reference genome for downstream analyses. Short reads from both parents and offspring were aligned to this custom reference using BWA-MEM2 (Vasimuddin et al. 2019). We then computed the read-depth distribution for each contig in both parents and offspring. An example is shown for a pair of paternal bubble contigs. Based on the read-depth distributions in parents and an offspring, we identified the inherited contig from the father in the offspring. The inheritance states of all parental contigs and read alignments were further used to identify DNMs in each offspring, with an exemplar DNM shown in the bottom right panel.

However, we reasoned that the high heterozygosity of *B. floridae* can facilitate allele-aware assembly of the parental genomes, as many divergent regions in the parental genomes can be assembled into separated contigs. The high sequencing depths of our data enabled us to do contig-level de novo assembly for two parents. Using the parental genome assembly as a custom reference, we should be able to align most offspring and parental reads to the parental genomes, thereby obtaining high-quality genotype information of offspring and parents for DNM detection (Fig. 1).

Therefore, we constructed the genome assembly for each parent using the allele-aware assembler Platanus-allee (v2.2.2) (Kajitani et al. 2019). We obtained a maternal genome assembly of 805.9 Mb and a paternal genome assembly of 830.0 Mb (Table S3), approximately 1.7 times the length of the previously published haploid genome (490 Mb) (Huang et al. 2023), indicating that a large fraction of the diploid parental genome was assembled into phased alleles. By the definition of Platanus-allee, heterozygous regions are assembled as “bubble” contigs with two allelic sequences, named as “primary” and “secondary,” respectively. Homozygous regions with identical or nearly identical sequences in an individual are assembled as “non-bubble” contigs (Fig. 1). We found that, owing to the limitations of Platanus-allee, non-bubble contigs—which, in principle, should derive exclusively from homozygous sequence—are in practice a mixture of homozygous and heterozygous sequences. Accurately distinguishing homozygous from heterozygous sequence is essential both for DNM discovery and for downstream analyses of DNMs. We therefore refined our pipeline for classifying non-bubble contigs (see Methods). By combining information from contig self-alignments and the genome-wide distribution of contig-level read depths, we distinguished true non-bubble contigs (homozygous sequence) from “bubble-like” contigs (heterozygous sequence), while designating the remainder as uncertain non-bubble contigs. Using this procedure, approximately half (55.93%) of the original non-bubble contigs were reclassified as heterozygous (Table S4), and simulation analyses (see Methods) indicate an overall classification accuracy of ∼95%, indicating that our treatment is reliable (Table S5).

By merging the two parental genome assemblies, we generated a composite parental reference genome, with detailed assembly statistics provided in Table S4. The parental reference genome had a total length of approximately 1.6 Gb, consisting of 263,358 bubble contigs covering ∼856 Mb and 758,976 non-bubble contigs spanning ∼780 Mb. The contig N50 metrics of maternal and paternal assemblies were 6.6 kb and 7.6 kb, respectively. More than 91% of sequencing reads of the two parents were aligned to this parental reference genome with proper insert sizes (Figure S1), indicating that the vast majority of genomic segments were successfully assembled and available for downstream analysis. We also evaluated both parental assemblies using BUSCO, a tool for assessing genome assembly and annotation completeness. We found that only ∼5% of core single-copy orthologs were classified as missing, whereas ∼28% were recovered in fragmented form, most likely reflecting the limited contiguity of our short-read–based assemblies (contig N50 < 10 kb) rather than true gene absence. The BUSCO analysis indicates that the assembled contigs have high gene coverage but relatively modest contiguity. We note that in our study the parental assemblies are used primarily as the alignment reference, and functional annotation is performed by aligning the parental contigs against the published reference genome.

**Figure 2:**
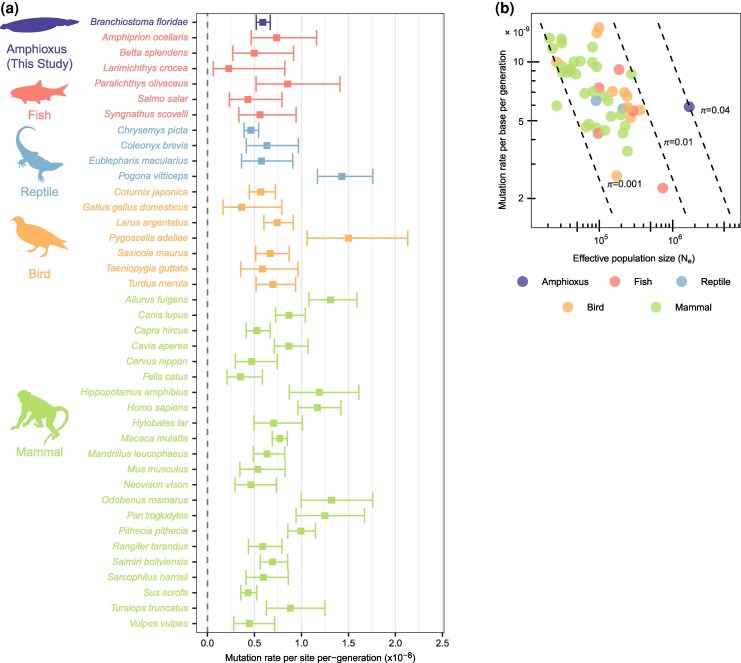
Estimates of mutation rate and effective population size. a) Per base per generation DNM rate (*μ*) estimates from this study and previous studies (Bergeron et al. 2023). Error bars denote 95% CIs. b) Comparison of mutation rates and effective population sizes across diverse taxonomic groups, using data for other taxa from Bergeron et al. (2023). The dashed lines from left to right represent *π* values of 0.001, 0.01, and 0.04, respectively. The *π* values, labeled near each dashed line, were calculated according to *π* = 4*N*_e_*μ*.

Next, we aligned sequencing reads from both offspring and parents to this parental reference genome and detected variants using BCFtools (v1.21) (Danecek et al. 2021) and freebayes (v1.3.10) (Garrison and Marth 2012). A custom filtering pipeline, accounting for factors such as read depth and variant quality, was employed to obtain high-confidence DNMs (see Methods). After applying the filtering criteria, 803 candidate DNMs were retained. Among these DNMs, putative false positives were further ruled out by manual check with IGV visualization (see Methods). After careful check, a total of 242 high-confidence DNMs (Table S6) were retained across 104 offspring, corresponding to an average of 2.33 DNMs per individual. Twenty-two DNMs from nine individuals were selected for orthogonal validation using independent amplicon-seq data. For each selected DNM, 150 to 200 bp regions encompassing the DNM were amplified and sequenced for both the offspring and parents. The amplicon-seq data confirmed 20 of the 22 selected DNMs as true DNMs (Tables S7 to S9), while the remaining two (in a same amplicon) yielded inconclusive results due to amplification noises, indicating a low false-positive rate in our DNMs. With an average callable genome size of 414.8 Mb and a false-negative rate (FNR) of 4.8% estimated by simulation (see Methods) (Table S10), the mean genome-wide mutation rate was calculated to be 5.89 × 10^−9^ mutations per base per generation, with a 95% confidence interval (CI) of 5.20 to 6.68 × 10^−9^. The mutation rate of *B. floridae* is comparable to those reported in fish and other vertebrates (Bergeron et al. 2023) (Fig. 2a).

### High heterozygosity of *B. floridae* is mainly attributed to its large effective population size

It has long been recognized that the genomic heterozygosity of *B. floridae* is much higher than in other species (Putnam et al. 2008). Yet, since the mutation rate in *B. floridae* has not been directly quantified, the cause of its high heterozygosity remains uncertain. The nucleotide diversity, expressed as *π*, can be represented by *π* = 4*N*_e_*μ* under neutral evolution (Nei and Tajima 1981; Kimura 1983), where *N*_e_ represents the effective population size and *μ* the germline mutation rate. Therefore, the effective population size can be estimated by the equation *N*_e_ = *π*/(4*μ*). Based on synonymous substitutions in the population genomic data from Huang et al. (2023), we obtained the *π* estimate of 0.0397 for *B. floridae* (Methods). For comparison, the *π* estimates of most vertebrate species from Bergeron et al. (2023) fall into the range of 0.001 to 0.01, with none above 0.01. The effective population size, calculated with our estimated mutation rate (*μ*) and *π*, was 1,684,375. Bergeron et al. (2023) also performed pairwise sequentially Markovian coalescent (PSMC) method (Li and Durbin 2011) to investigate *N*_e_ dynamics and reported that PSMC-based harmonic mean *N*_e_ estimates are generally consistent with those estimated from *π*/(4*μ*) across species. Using the same pipeline as Bergeron et al. (2023), we obtained a harmonic mean *N*_e_ of approximately 300,000 for the parental individuals (Figure S4)—substantially lower than the *N*_e_ derived from the formula *π*/(4*μ*). Upon further inspection, we determined that this discrepancy stems primarily from the low number of heterozygous sites detected during the alignment step, a known technical challenge when analyzing highly heterozygous genomes (see Methods). Consequently, for cross-species comparisons, we used the *N*_e_ for *B. floridae* derived from the formula *π*/(4*μ*).

Despite the underestimated *N*_e_ values, the temporal trends inferred from the PSMC analysis remained informative. For the past 1 million years, the *N*_e_ trajectories of the two parental individuals (Figure S4) peaked around 300,000 to 200,000 years ago, followed by a mild decline between 200,000 and 50,000 years ago, and a rapid decrease beginning approximately 30,000 years ago. The most recent decline in the PSMC trajectory could be linked to the Last Glacial Maximum occurring ∼26,500 to 19,000 years ago (Clark et al. 2009) and/or may partially reflect laboratory inbreeding.

We placed *B. floridae*'s *N*_e_ and *μ* within the context of *N*_e_ and *μ* values derived from Bergeron et al. (2023) to show its unique position (Fig. 2b; Table S10), indicating that *B. floridae*'s *N*_e_ is largest among chordates with measurements. We note that the sequenced population in Huang et al. (2023) experienced some degree of inbreeding in the laboratory, which could lead to lower nucleotide diversity than wild populations. Therefore, the actual *N*_e_ in wild populations is likely larger than the current estimate. Moreover, the recent bottleneck inferred by PSMC would further reduce nucleotide diversity, suggesting that historical effective population sizes were even larger in the past. Collectively, we concluded that the high heterozygosity of amphioxus is not due to an elevated mutation rate but a very large effective population size.

### Characteristics of DNMs in *B. floridae*

**Figure 3:**
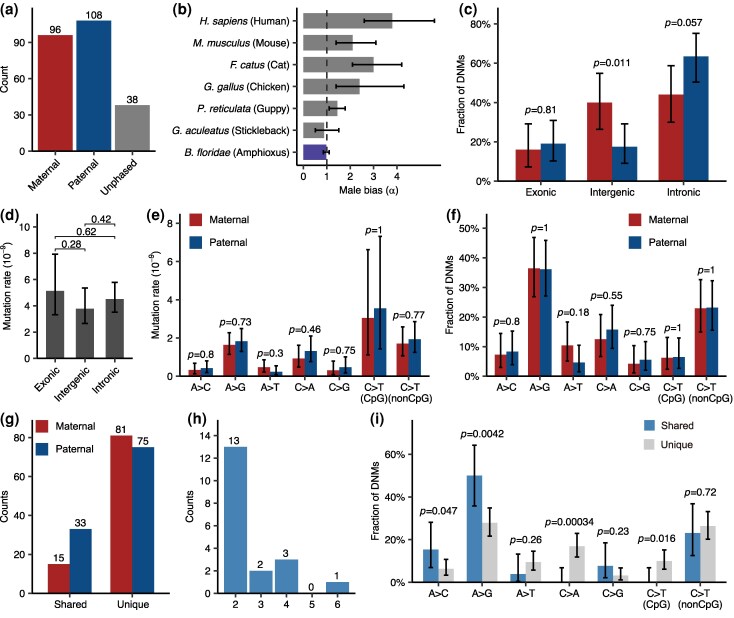
Characteristics of DNMs in *B. floridae*. a) Parental origins of DNMs. b) The male-to-female ratios (α) of DNMs for different chordates. c) Proportions of DNMs in different gene annotation contexts, separating paternal and maternal DNMs. Parental origin differences were assessed by Fisher's exact tests. d) Mutation rates of different gene annotation contexts. Pairwise comparisons of mutation rates among gene annotation contexts were performed using χ² tests. e) Mutation rates of different substitution types, separating paternal and maternal DNMs. Parental origin differences assessed by χ² tests. The mutation spectrum was folded (e.g. A>C representing both A>C and T>G mutations). f) Proportions of DNMs of different substitution types, separating paternal and maternal DNMs. Parental origin differences were assessed by Fisher's exact tests. g) Counts of sibling-shared and unique DNMs, separating paternal and maternal DNMs. h) Counts of sibling-shared DNMs shared by different numbers of offspring. i) Proportions of DNMs of different substitution types, separating shared and unique DNMs. Differences between shared and unique DNMs were assessed by Fisher's exact tests. Error bars denote 95% CIs computed using binomial distributions. All *P*-values for Fisher's exact tests and χ² tests are given above the bars.

Distinguishing the parental origins of DNMs is crucial for understanding mutational dynamics. While previous studies could only assign parental origin for 30% to 80% of DNMs based on nearby polymorphic markers (Bergeron et al. 2023; Wooldridge et al. 2025), our DNM calling method provided parental origin information for most (84.3%) detected DNMs. Among the 204 phased DNMs, 96 originated from the mother and 108 from the father (Fig. 3a), yielding a male mutation bias coefficient (α) of 1.12. Considering the difference in the callable genome sizes between the parents, we obtained a corrected α of 0.98 (95% CI = 0.85 to 1.10), indicating a slight maternal bias. For a cross-species comparison, we integrated several representative vertebrates with known α values (Fig. 3b).

Due to the fragmented nature of the parental genome assemblies, direct genomic annotation was challenging; therefore, we mapped the parental genome coordinates to the published haploid reference genome from Huang *et al*. (Huang et al. 2023) and used its annotation for downstream functional analyses. Of the total 490 Mb of the public haploid reference genome, 363 Mb (74%) was covered by the assembled contigs of parental genomes. The parental contigs that were mapped to the haploid reference genome were defined as mappable regions with annotation information. Based on the haploid genome annotation (Huang et al. 2023), the genome was divided into three gene annotation contexts: exonic, intronic, and intergenic regions. We found no significant difference between the fractions of paternal and maternal DNMs for each gene annotation context (Fig. 3c). We also compared mutation rates across different gene annotations and found no significant differences among them (Fig. 3d). We note that the greater evolutionary conservation of exonic sequences enables a larger proportion of them (assembly: 11.86% exonic; mapped: 13.46% exonic) to be confidently aligned to the published reference genome compared to sequences in other annotation contexts. Mapping bias could compromise the representativeness of the comparison across annotation contexts.

We further investigated DNM counts of different substitution types. The results indicated no significant difference between maternal and paternal mutation rates for all substitution types (Fig. 3e) and no significant difference between parental origins when looking at proportions of DNMs of different substitution types (Fig. 3f). In vertebrates, the mutation rate of C>T at CpG sites is usually much higher than that at non-CpG sites (e.g. >10-fold difference in humans) (Kong et al. 2012; Francioli et al. 2015). However, the mutation rate of C>T at CpG sites was only about twice that at non-CpG sites in *B. floridae* (Fig. 3e). Cross-species comparison of mutational spectra (Figure S5) revealed some unique patterns in *B. floridae*. CpG>TpG mutations only accounted for 8% of DNMs in *B. floridae*, significantly lower than those (16% to 20%) observed in vertebrates. On the other hand, A>G mutations accounted for 33% of DNMs in *B. floridae*, consistently higher than those (16% to 26%) in surveyed vertebrates. The lower mutation frequency at CpG sites was probably due to lower methylation levels at CpG sites in amphioxus (Marletaz et al. 2018).

Fifty-two (21%) DNMs were shared among siblings (named ssDNMs, Fig. 3g), which likely occurred before specification of primordial germ cells (PGCs) or during early PGC proliferation, allowing the same mutation being transmitted to several full-sibling offspring (Jonsson et al. 2018). A greater number of ssDNMs were identified as paternal in origin, with 33 ssDNMs derived from the father compared to only 15 from the mother, and the remaining 4 ssDNMs were unphased. The male bias α of 1.91 (after accounting for callable size difference) in ssDNMs was substantially higher than the α of 0.98 observed for all DNMs (Fisher's exact test, *P* = 0.021), suggesting that during earlier stages of germ cell development, male germline accumulates more mutations relative to female germline. Majority of ssDNMs were shared by only two offspring, with one shared by up to six offspring (Fig. 3h). Comparison of mutation spectra indicated a significant increase in the proportion of A>G mutations in ssDNMs relative to unique DNMs, but a depletion of C>A mutations in ssDNMs, likely reflecting the differences in mutational processes between early and late gametogenesis (Fig. 3i).

We also examined the contigs and DNMs mapped to the sex-determining region (SDR, ∼5 Mb) reported in *B. floridae* (Huang et al. 2023). The contigs in our assemblies that can be aligned to the SDR span 1.2 Mb in the maternal genome and 1.1 Mb in the paternal genome. We did not detect any DNM within this region. Given the low mutation rate and small SDR size, the absence of DNMs in the SDR is not surprising.

### Characteristics of PZMs in *B. floridae*

**Figure 4:**
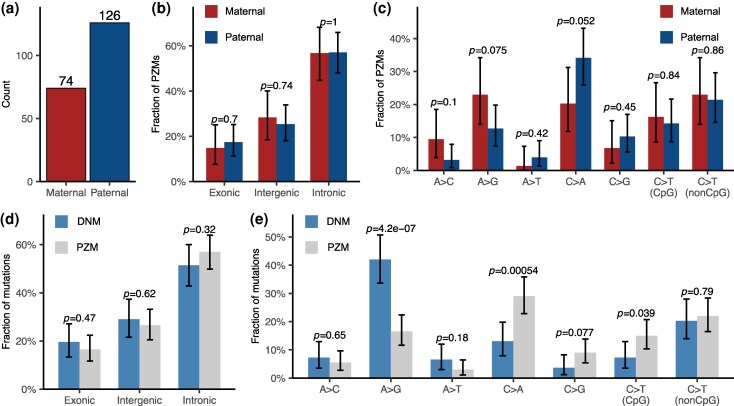
Characteristics of PZMs. a) Parental origins of PZMs. b) Proportions of PZMs in different gene annotation contexts, separating paternal and maternal PZMs. Parental origin differences were assessed by Fisher's exact tests. c) Proportions of PZMs of different substitution types, separating paternal and maternal PZMs. d) Proportions of mutations in different gene annotation contexts, separating DNMs and PZMs. e) Proportions of mutations of different substitution types, separating DNMs and PZMs. Parental origin differences and differences between DNMs and PZMs were assessed by Fisher's exact tests, with *P*-values above the bars. Error bars denote 95% CIs computed based on binomial distributions.

PZMs arise during mitotic cell divisions in the developing embryo or later in life and thus typically affect only a subset of the body's cells. Some PZMs are present in the germ cells can be passed to the next generation. The high sequencing depths of our data also enabled us to detect PZMs in offspring (see Methods for details). We identified 200 putative PZMs across 104 offspring (Fig. 4a; Table S11), yielding an average of 1.92 PZMs per individual. The variant allele frequencies (VAF) of PZMs ranged from 0.05 to 0.36 (Table S11). As the detected PZMs had relatively high allele frequencies, they probably arose during early development.

Unexpectedly, PZMs in *B. floridae* exhibited a pronounced paternal-origin bias (α = 1.48, corrected for callable size difference; binomial test, *P* = 1.45 × 10^−4^; Fig. 4a). We further examined differences in parental origin with respect to proportions of PZMs across gene annotation contexts and across mutation types, but found no significant difference (Fig. 4b and c). Similarly, comparison between PZMs and DNMs in gene annotation contexts revealed no notable difference (Fig. 4d). However, analysis of specific mutation types revealed that A>G mutations were significantly more frequent in DNMs than in PZMs. Conversely, C>A mutations were markedly enriched in PZMs relative to DNMs, and C>G and CpG>TpG mutations were also more prevalent in PZMs (Fig. 4e). While the parental origin ratio of PZMs was close to that of ssDNMs (PZM: 1.48; ssDNM: 1.91), their mutation spectra were largely different (Fig. 3i versus Fig. 4e), suggesting different underlying mutational mechanisms.

## Discussion

In this study, we generated high-depth short-read sequencing data for a pedigree comprising both parents and 104 offspring to investigate DNMs in amphioxus. Due to high heterozygosity of amphioxus, which results in many sequence differences between the public reference genome and re-sequenced individuals, conventional alignment and variant calling methods that rely on the public reference genome are prone to errors, thereby complicating DNM detection. However, high heterozygosity can also facilitate allele-aware diploid genome assembly without requiring long-read data. With this consideration, we assembled haplotype-resolved diploid genomes at the contig level for each parent and developed a custom read mapping and variant calling pipeline using the parental assemblies as the reference. This cost-effective strategy can help resolve the challenges of detecting DNMs in highly heterozygous species like *B. floridae*. It also allowed us to assign the parent-of-origin state for most detected DNMs. Furthermore, based on phased genotypes of parents and offspring, we were also able to comprehensively investigate the meiotic recombination landscape, which has been described in a companion study (Tao et al. 2025).

With an average callable diploid genome size of 414.8 Mb, we identified 242 DNMs and estimated the germline mutation rate in *B. floridae* to be 5.89 × 10^−9^ (95% CI of 5.20 to 6.68 × 10^−9^) per base per generation. Based on this mutation rate, the effective population size of *B. floridae* was estimated to be about 1.7 million, suggesting that the high heterozygosity resulted from a massive effective population size.

Apart from the large effective population size, other factors such as hybridization, gene flow, recombination, and demographic history may also contribute to high heterozygosity. Hybridization and gene flow cannot be assessed with our current pedigree dataset, and thus, we are unable to evaluate their contributions. Recombination was examined in a companion study, which showed that multiple features of amphioxus recombination resemble those in vertebrates. And there is no evidence that unusual recombination patterns can explain the high heterozygosity in this species. With respect to demographic history, we inferred *N*_e_ dynamics using PSMC based on heterozygous sites identified by mapping our reads to the public reference genome. Due to alignment challenges with high sequence difference, the observed heterozygosity (∼0.6%) using this conventional method is roughly 20% of the expected level as estimated by *k*-mer analysis. Under this conservative estimate, the harmonic mean *N*_e_ between 30,000 and 1,000,000 years ago is approximately 300,000. If heterozygosity were closer to its true level, we anticipate that the corresponding harmonic mean *N*_e_ would likely exceed 1 million, further supporting a large long-term *N*_e_ for this lineage. In summary, we propose that a large effective population size is the primary driver of the high heterozygosity, though the potential influence of other factors merits future study.

Our estimated mutation rate for contemporary populations is close to a recently reported estimate (4.65 × 10^−9^) based on divergence times and intra-cephalochordate synonymous substitution rates of single-copy genes (Ren et al. 2025). Our study did not estimate the germline mutation rate for small insertions/deletions (INDELs), as generally poor alignments around these variants (usually associated with repeats) make it challenging to accurately detect de novo INDELs. In addition, repetitive or low-complexity regions, which usually have relatively high mutation rates, are difficult to assemble with only short reads and thus were under-represented in the callable regions of our study. Future studies incorporating long-read sequencing technologies (e.g. PacBio or Oxford Nanopore) can facilitate identifying de novo INDELs as well as other DNMs in complex genomic regions.

We further analyzed the parental origin, genomic distribution, mutation spectrum, and ssDNMs in *B. floridae*, revealing some distinctive features in this basal chordate. We observed a maternal bias of DNMs, with a male-to-female mutation ratio (α) of 0.98. This ratio was lower than most reported values for vertebrates. Previous studies (de Manuel et al. 2022; Bergeron et al. 2023; Zhang et al. 2025) reported that fish tend to have apparently lower α values than other vertebrates (mammals, birds, and reptiles). These studies suggested that differences in gametogenesis such as seasonal breeding and synchronous ovulation could lead to the lower α values in fish. The low α of amphioxus might be partly due to these reasons.

The overall mutation spectra showed no obvious difference between different parental origins. The low frequency of C>T transitions at CpG sites was consistent with previously reported low levels of CpG methylation in amphioxus (Marletaz et al. 2018). The presence of ssDNMs provided a valuable window into early germline mutational events. We observed more ssDNMs in the paternal genome relative to the maternal, but the underlying cause is unclear. Future DNM studies spanning different ages and developmental stages of parents can help clarify the mechanisms of mutagenesis in amphioxus. In addition, functional genomics studies focusing on DNA damage and repair pathways are also essential to understanding the mutational mechanisms. We also note that the sequenced strain underwent several rounds of laboratory inbreeding; whether this has an effect on mutation rate warrants further investigation.

We identified 200 putative PZMs across 104 offspring, with a surprisingly strong paternal-origin bias (α = 1.48). The sex-biased pattern of PZMs, which has not been observed in other species to our knowledge, suggested different mutational processes operating on paternal and maternal chromosomes during early development. In mammals, early divisions of embryos erase epigenetic memory (primarily DNA methylation and histone modifications) from sperm and egg and reestablish the epigenomic environment. Yet in some vertebrates such as zebrafish and sea lamprey (Potok et al. 2013; Angeloni et al. 2024), the methylation landscape in the paternal genome of early embryos is largely maintained and the maternal methylation is reprogrammed to match the paternal. Available methylome data suggested that similar patterns are likely to be present in sea squirt and sea urchin (Xu et al. 2019). While there was no study comparing methylomes of gametes and early embryos in amphioxus, we suspected that amphioxus may employ similar epigenetic reprogramming like that in sea lamprey, given its phylogenetic position. The epigenomic differences between paternal and maternal chromosomes in early embryos might contribute to different frequencies of paternal and maternal PZMs. Further functional genomic analysis is needed to test this hypothesis.

While PZMs and DNMs showed similar distributions across gene annotation contexts, PZMs were significantly enriched for C>A and CpG>TpG mutations, whereas germline DNMs more frequently involved A>G mutations. As most PZMs are somatic mutations, the distinct mutation spectra between PZMs and DNMs highlighted different biological mechanisms governing mutagenesis in somatic versus germline lineages. Previous methylome analysis revealed that amphioxus embryos exhibit more methylated CpGs relative to adult tissues, which may partly explain the relatively higher frequency of CpG>TpG mutations in PZMs (Marletaz et al. 2018). The COSMIC mutational signatures SBS1 and SBS5 have been reported as the two primary contributors to germline mutations in mammals (Beichman et al. 2023). SBS1 has a known cause—the deamination of methylated cytosine—leading to C>T mutations at CpG sites. SBS5, though etiologically unclear, is thought to reflect a ubiquitous, clock-like endogenous mutational process. SBS5 exhibits a relatively high frequency of A>G mutations. The elevated proportion of A>G mutations observed in amphioxus DNMs may reflect a greater contribution from SBS5 compared to SBS1. However, due to the limited number of DNMs detected in this study, we were unable to perform a robust mutational signature analysis to test this hypothesis.

In summary, through high-depth pedigree sequencing and the development of a novel analysis pipeline, we directly quantified the germline mutation rate in *B. floridae* for the first time and proposed that the large effective population size is the primary driver of the observed high heterozygosity. We also explored DNMs from multiple perspectives, including parental origin, gene annotation context, mutation spectrum, and shared DNMs among siblings. Some of the unique DNM features observed in amphioxus may represent ancestral characteristics of germline mutagenesis in chordates. The analysis framework based on assembling parental genomes for read alignment and variant calling can facilitate related research in other highly heterozygous species.

## Materials and methods

### Amphioxus culture

The amphioxus strains used in this study originated from a founder population of approximately 30 individuals collected from seawater near Tampa, Florida, and were maintained by Dr. Jr-Kai Yu. Following two generations of inbreeding, about 100 offspring were transferred to the laboratory at Xiamen University and reared under previously described conditions (Li et al. 2012). After approximately four additional rounds of inbreeding in Xiamen laboratory, one pair of parental individuals and 104 F1 offspring derived from this pair were selected for the study. To capture distinct developmental time points, the offspring were snap-frozen in liquid nitrogen at two specific post-growth intervals: 6 months and 1 year. The first batch, comprising 35 individuals, was preserved at the 6-month time point, and the second batch, consisting of 71 individuals, was preserved at the 1-year time point.

### Library construction and sequencing

We extracted high molecular weight genomic DNAs from the muscle tissues of a single individual using the DNeasy Blood & Tissue Kit (QIAGEN, Valencia, CA) and inspected the DNA quality by Qubit 2.0 Fluorometer (Thermo Fisher Scientific, Waltham, MA) and 2100 Agilent Bioanalyzer (Agilent). After sample QC, gDNA was fragmented by ultrasound on Covaris E220 (Covaris, Brighton, United Kingdom), then selected to 300 to 500 bp using magnetic beads size selection. The selected DNA fragments were then end-repaired and A-tailed. The indexed adaptors were ligated to both ends of the DNA fragments. The ligation product was amplified by PCR and circularized to get single-stranded circular (ssCir) library. The ssCir library was then amplified through rolling circle amplification (RCA) to obtain DNA nanoball (DNB). The DNB was then loaded to flowcell and sequenced by DNBSEQ Platform (MGI, China).

### Parental genome assembly

Genome heterozygosity of parental individuals was estimated via *k*-mer analysis of Illumina short-read data using GenomeScope (v2.0) (Ranallo-Benavidez et al. 2020). Raw short-read sequences were quality-processed with fastp (v0.24.0) (Chen 2023), implementing (i) elimination of reads with >10% ambiguous bases (‘N’) and (ii) retention of reads exhibiting a minimum mean Phred quality score of 20 (Q20). Adapter sequences were concurrently trimmed. These processed reads were subjected to allele-aware diploid genome assembly using Platanus-allee (v2.2.2) (Kajitani et al. 2019) through a two-stage workflow: First, the de novo assembly phase was executed with the command “assemble -m 200 -t 20”; second, haplotype resolution was performed via the phase module using “phase -i 3 -t 20.” Contigs shorter than 150 bp in length were subsequently filtered from both parental phased assemblies. Initial assembly quality metrics, including contig N50, total length, and GC content, were comprehensively evaluated with QUAST (v5.3.0) (Gurevich et al. 2013) and SeqKit (v2.9.0) (Shen et al. 2016).

Following the phased assembly procedure, contigs underwent automated classification by Platanus-allee. In the contig-level assemblies generated by Platanus-allee, contigs were classified into two distinct categories: (i) bubble contigs harboring allelic variants, which were further stratified as primary contigs (longer allelic sequences) and secondary contigs (shorter counterparts), and (ii) non-bubble contigs designated by the assembler as lacking allelic diversity. However, through comprehensive whole-genome alignment analyses, we discovered that a subset of non-bubble contigs exhibited unexpected sequence overlaps with other contigs across the assembly, suggesting residual heterozygous regions unresolved by the phasing algorithm.

Finally, the filtered phased diploid contig sets derived from paternal and maternal genomes were concatenated to construct a custom reference genome (hereafter termed parental reference genome), which was used in many downstream analyses.

### Reclassification of non-bubble contigs

In genome assemblies, the distinction between heterozygous and homozygous contigs typically relies on two complementary sources of information: (i) self-alignment among contigs and (ii) the depth distribution of short reads aligned back to the assembly. We combined these two lines of evidence to assign zygosity to each contig. Conceptually, this strategy is related to tools such as purge_dups (Guan et al. 2020) and Redundans (Pryszcz and Gabaldon 2016), which also combine read depth and sequence similarity to distinguish primary contigs from haplotigs in highly heterozygous assemblies. For clarity, we describe the procedure for the maternal assembly, noting that the same strategy was applied to the paternal assembly.

Maternal short reads were mapped to the newly assembled maternal reference genome using BWA-MEM2 (v2.2.1) (Vasimuddin et al. 2019). We then computed the mean coverage of each contig by averaging the per-base depth across its length, yielding a genome-wide distribution of contig-level mean depth. As expected for a diploid genome, this distribution was clearly bimodal: the higher-depth peak corresponds to homozygous contigs, whereas the lower-depth peak corresponds to heterozygous contigs for which only one haplotype is represented in the contig sequence. We exploited this bimodal depth distribution to define thresholds for heterozygous and homozygous contigs.

Based on the bimodal distribution of mean read depths of assembled contigs (as shown in Figure S6), we decided the depth ranges for bubble-like contigs and true non-bubble contigs in the original non-bubble contig set. Accordingly, we chose a depth range of 10 to 50× as the filtering criterion for bubble and bubble-like contigs (Figure S6). For obtaining true non-bubble contigs, we chose a depth range of >50× and ≤100× (Figure S6). Using these depth thresholds, we roughly partitioned all non-bubble contigs into candidate heterozygous and candidate homozygous sets, which were further refined in subsequent steps.

To more precisely establish homology relationships among contigs, we performed all-vs-all self-alignment with Minimap2 (v2.24) (Li 2021), with parameters: “-ax asm20 –secondary = no.” To reduce artifacts from short contigs and spurious local matches, alignments were filtered based on both alignment coverage and sequence identity before identifying reciprocal best hits (RBHs); only pairs with values ≥ 0.7 for both metrics were retained. We considered the hallmark of a heterozygous contig to be the presence of a high-quality RBH to another contig in the assembly. Accordingly, two candidate heterozygous contigs that formed an RBH pair in the self-alignment were designated as “bubble-like” contigs and grouped into a bubble-like pair. These bubble-like contigs were subsequently treated as bubble contigs in all downstream analyses.

For candidate homozygous contigs, we applied an analogous RBH-based filtering strategy in a “negative list” fashion. Unlike the heterozygous case, where RBHs are searched only among candidate heterozygous contigs, each candidate homozygous contig was screened for RBHs against all other contigs. If an RBH satisfying our coverage and identity filters was found, the focal contig was considered potentially heterozygous and removed from the homozygous set. Only contigs with no qualifying RBH to any other contig were retained as true non-bubble (homozygous) contigs. All remaining non-bubble contigs that were neither grouped into bubble-like pairs nor retained as true non-bubble contigs were designated as uncertain non-bubble contigs.

### Assessing the mis-classification rate of non-bubble contigs

To quantify the accuracy of our classification scheme, we adopted a validation strategy that combines internal positive controls with simulation-based benchmarking. As noted above, assembler-defined bubble contigs are expected to be highly reliable representations of heterozygous sequence and therefore provide a natural positive control for evaluating the bubble-like category. Specifically, during identification of bubble-like contigs, we subjected the original bubble contigs to the same RBH-based pipeline and quantified the fraction that remained classified as heterozygous after filtering; this allowed us to estimate the accuracy of the bubble-like classification.

In parallel, we independently validated the method's performance using simulated data. We simulated diploid genomes with spatially variable heterozygous SNP density using a window-based inhomogeneous Poisson approach. The reference genome was divided into non-overlapping windows, and simulated SNPs were counted per window. Empirical SNP counts per window, obtained from a published population variant file (Huang et al. 2023), were used to define window weights. Given a target total SNP number from a desired heterozygosity, we allocated SNPs to windows in proportion to these weights using independent Poisson sampling. Within each window, SNP positions were drawn uniformly from simulated SNP while excluding a set of randomly placed non-overlapping “no-variant” intervals. At each selected site, a heterozygous SNP was introduced by mutating one randomly chosen haplotype, yielding two haplotype FASTA files and a corresponding VCF.

We randomly selected one of the longer amphioxus chromosomes and introduced heterozygous SNPs as described above to obtain a diploid template with two haplotypes (hap1 and hap2). Short-read data were then simulated at 80× haploid coverage from each haplotype using ART and assembled with the same pipeline as for the real data as described in section “Parental genome assembly,” yielding a mixture of bubble and non-bubble contigs. To distinguish whether assembled contigs from the simulated reads originated from no-variant or other parts of the simulated genome, we aligned all contigs back to the simulated reference. Contigs mapping to predefined no-variant regions were classified as true non-bubble contigs, whereas those mapping elsewhere were classified as bubble-like contigs. These labels were treated as ground truth to evaluate the accuracy of our pipeline for classifying non-bubble contig categories.

### Alignment of offspring and parental reads to the parental reference genome and variant calling

Processed short reads from two parents and offspring were aligned to the parental reference genome assembly using BWA-MEM2 (v2.2.1) (Vasimuddin et al. 2019) with parameters “-M -k 19.” Post-alignment processing entailed coordinate-based sorting and removal of PCR duplicates via SAMtools (v1.21) (Li et al. 2009) and Picard Tools in GATK4 (v4.6.1.0) (Van der Auwera and O’Connor 2020) with default parameters. For the multi-hit alignments, one randomly chosen alignment was retained using Sambamba (v0.8.2) (Tarasov et al. 2015). Per-base depths across the whole reference genome and individual-level average sequencing depth metrics were subsequently computed using mosdepth (v0.3.10) (Pedersen and Quinlan 2018) with default parameters.

Based on the read alignments above, we used BCFtools (v1.21) (Danecek et al. 2021) and freebayes (v1.3.10) (Garrison and Marth 2012) to identify small variants. First, we generated “pileup” files for the set of all samples by running “bcftools mpileup” with parameters “-annotate FORMAT/AD, FORMAT/ADF, FORMAT/ADR, FORMAT/DP, FORMAT/SP, INFO/AD, INFO/ADF, INFO/ADR” on all input bam files. With the “pileup” files as input, we ran “bcftools call -mv” to generate VCF files, which contain SNVs and short INDELs, for 104 offspring and two parents. We additionally used freebayes (v1.3.10) (Garrison and Marth 2012) to identify variants within similar sequences between the two parents with parameters “-m 0 –legacy-gls.” In regions exhibiting high sequence similarity between the two parents, only the freebayes calls were retained; in all other regions, variant calls from both tools were used.

### Inferring inheritance states of parental contigs in offspring genomes based on read alignments against the parental reference genome

According to the principles of Mendelian inheritance, genomic reads in the offspring are derived from the gene pool of two parental genomes. As described in previous sections, we obtained the paternal and maternal diploid genome assemblies as sets of haplotype-resolved contigs. By mapping sequencing reads of an offspring back to the parental reference genome (combining paternal and maternal genome assemblies), we can determine which allele of a specific locus (bubble or non-bubble contigs) of a given parent is inherited by the offspring. More specifically, to determine whether a particular contig from a given parent is inherited by a specific offspring, we compared read-depth distribution profiles of that contig between the offspring and each parent. For a contig with specific parental origin, the depth distribution of the offspring's reads aligned to this contig is expected to more closely resemble the depth distribution of the reads from the parent-of-origin rather than that from the opposite parent.

We divided inheritance inference into two stages, depending on whether parental allelic information is incorporated. In “stage 1,” inheritance was inferred for each contig independently, based solely on its read-depth distribution (Figure S7). In “stage 2,” these assignments were refined by jointly considering the similarity of read-depth distributions between the two parents and the expected inheritance patterns of allelic contigs within and across parents (Figures S8 and S9).

The stage 1 procedure is described below and illustrated in Figure S7. To quantify the depth distribution along a contig, each contig was divided into 50 bp bins, discarding any terminal bin shorter than 50 bp. The mean depth within each bin was calculated for each individual, generating a depth distribution vector per individual across the contig. The similarity between offspring and parental vectors was quantified using a weighted measure that combines (i) cosine similarity for bins with non-zero depth in both individuals being compared and (ii) Jaccard similarity for bins with zero coverage in at least one of the two individuals (accounting for regions of no aligned reads). Weights of different similarity scores were allocated based on corresponding proportions of zero-value bins and non-zero-value bins. A parental contig was considered inherited by an offspring if its depth distribution vector in that offspring showed greater similarity to the vector of its specific parental source than to the other parent. Parental contigs exhibiting a depth less than 1/4 of an offspring's mean depth or a depth less than 10 were classified as low-depth and thus non-inherited in the specific offspring.

However, the similarity of some contigs between the parental genomes, probably arising from shared ancestry, could confound the inheritance calls derived from the offspring-parent depth distribution similarity algorithm (Figure S7). Since direct pairwise genome sequence alignment of two parental genomes is difficult for assessing parental contig similarities due to potential length disparities of orthologous contigs in different parental assemblies, we used the similarity of read-depth distributions (calculated using the same algorithm employed for offspring-parent comparisons, named “parental similarity score”) when mapping each parent's reads to the same contig as a robust proxy. If the parental similarity score for a specific contig is greater than 0.5, the inheritance calls for that contig in the offspring are deemed unreliable due to indistinguishable parental origins, necessitating further validation with additional data.

Stage 2 is therefore primarily designed to improve inheritance inference in contigs with parental similarity and to resolve potential inference conflicts between parental allelic contigs that would otherwise appear to violate Mendelian segregation (as shown in Figures S8 and S9). First, all true non-bubble contigs, which represent homozygous sequence in the parent, were by definition considered to be inherited by offspring. Because a given diploid offspring can inherit only one allele from each parent at a locus, determining the inheritance status of one contig within a pair of bubble contigs from a parent inherently resolves the inheritance status of both contigs in the pair. In other words, the inheritance of one contig in a bubble pair can be inferred based on the inheritance of its counterpart. Therefore, we further took advantage of the very low read depths in some loci to conclude that an allele was not inherited; under such circumstances, the alternative allele with aligned reads must be inherited by the offspring (scenarios 1, 2, 4, 5, 6 in Figure S9).

If both contigs passed the depth filter, we instead relied on the parental similarity score described previously. When only one contig in the pair exhibited low parental similarity score and was initially classified as inherited in stage 1, we accepted this assignment and designated its partner as not inherited. When both contigs were initially classified as inherited (both showing low parental similarity score) in stage 1, we retained the call for the contig with the lower parental similarity score and reclassified its partner as not inherited (scenarios 1, 2, 3 in Figure S9). The rationale behind this decision is: if the offspring inherited its partner contig (termed contig B), then most reads currently mapped to the focal contig (termed contig A) should have been more likely mapped to the other parental allele with relatively high similarity with contig B. In that case, the contig A would be expected to have very few mapped reads and might not have passed the read-depth filter. Conversely, when both contigs were initially labeled as not inherited, we forced inheritance of the contig with the lower parental similarity score and kept its partner as not inherited, thereby ensuring that exactly one contig per bubble pair was ultimately assigned as inherited.

If both contigs in a bubble pair exhibited high parental similarity, this indicated that each contig was identical, or nearly so, to the corresponding allele in the other parent, and the inheritance state of the pair could not be resolved from the available data (scenario 5 in Figure S9). Mutations arising on such contigs can still be detected, but they must be classified as unphased DNMs. For uncertain non-bubble contigs, inheritance could only be evaluated based on the similarity score. Contigs with similarity above the threshold were treated as uninformative and excluded from subsequent analyses, whereas contigs with similarity below the threshold were retained together with their inferred inheritance state. For completeness, all possible combinations of inheritance status and similarity class, and the resulting read-depth patterns in the offspring, are summarized in Figure S9.

Based on the inheritance states of parental contigs in each offspring, we in fact have reconstructed the offspring genomes with phased alleles in the majority of regions. Such phased offspring genomes can facilitate downstream analyses.

### Assessing the accuracy of inheritance inference

To evaluate the accuracy of our contig inheritance inference in offspring, we used the simulated diploid parental genomes described in the section “Assessing the accuracy of non-bubble contig classification.” For each parent, we randomly designated one haplotype (hap1 or hap2) as the inherited haplotig to the offspring, thereby defining a simulated diploid offspring genome. The short reads previously simulated from the two parental diploid genomes were reused as parental read data, and the corresponding contig-level parental assemblies served as parental reference.

We then aligned all contigs assembled with parental reads to the concatenated hap1 + hap2 reference using Minimap2 (v2.24) (Li 2021) while retaining secondary alignments. In this setting, contigs from heterozygous regions typically yield a single best alignment to one haplotype, whereas contigs from non-variant regions produce two equally good hits (one to each haplotype). For the purpose of defining ground-truth inheritance labels, we retained only contigs that aligned to the pre-designated inherited haplotig during simulation. We then applied our full inheritance-inference pipeline with simulated offspring reads and the combined parental reference (the concatenated maternal and paternal assemblies) and inferred, for each parental contig, whether it was transmitted to the simulated offspring. Comparison between inferred and true labels provided an empirical estimate of the accuracy of our inheritance assignments. Finally, using this approach, we estimated an error rate of ∼3% (Table S12), indicating that our method is reliable.

### Determining the callable genome

Estimating mutation rates requires determining the length of genomic regions within each offspring in which DNMs can be confidently identified, defined as the callable genome size. In each offspring, the callable genome is primarily composed of the inherited genomic segments defined in the previous section. For uncertain non-bubble contigs lacking preexisting allelic data in the parental genomes, the allele-depth-distribution-based method for inferring contig inheritance patterns is inapplicable. Consequently, determining the inheritance of homologous contigs within uncertain non-bubble contigs becomes challenging. We therefore implemented stricter filtering criteria, excluding uncertain non-bubble contigs with parental depth distribution similarity > 0.5 from the callable genome regions.

Regions with high sequence similarity between parental alleles impede the variant detection and phasing in our pipeline. In this study, the parents were derived from an inbred population, and thus, their offspring could inherit highly similar or identical alleles from parents in some regions. To address this, we developed a pipeline to identify pairs of parental contigs with high sequence similarity. Briefly, we performed pairwise genome alignments using Minimap2 (v2.24) (Li 2021) with parameters “-cx asm20 –secondary = no,” designating the paternal genome as the reference and the maternal genome as the query, and vice versa. RBH contig pairs were taken as evidence of homology. For each pair, we further assessed the parental similarity score; a pair of contigs with a parental similarity score > 0.5 was classified as a highly similar allele pair. For each offspring, if both contigs of a highly similar allele pair were inherited by the offspring, they were excluded from the callable genome for that offspring, as DNMs in these contigs could not be reliably phased and could be confounded by PZMs. In contrast, when only one homologous contig from either parent of a highly similar allele pair was inherited (scenario 5 in Figure S9), the contig was retained in the callable genome with the freebayes-called variants. For these unphased contigs, the parental origin of the inherited allele cannot be determined. However, we know that each offspring inherits exactly one of the two homologous alleles, which typically have comparable sequence lengths. Therefore, we approximate the callable genome size for unphased contigs in each offspring as half the total length of all unphased contigs. If a contig has a parental similarity score > 0.5 but no homologous contig can be identified in the other parent, it is also excluded, because in this situation we cannot determine whether the corresponding allele in the other parent has been inherited to the same offspring, irrespective of whether it is a bubble or a non-bubble contig.

Additionally, we removed sites in repetitive regions, as they are prone to erroneous alignments and often exhibit abnormally high alignment depths. Sites with too many reads may represent problematic repetitive regions (Li 2014). Specifically, based on the observed depth distribution in each offspring, any regions with a read depth exceeding three times the individual's average were excluded. The terminal regions of contigs (100 bp at each end) were also excluded from the callable genome, because these regions tended to enrich assembly and alignment errors.

Finally, by integrating these filters based on both inheritance and sequencing depth, we obtained the callable genome size for each offspring ranging from 334.09 to 499.26 Mb, accounting for approximately 34% to 51% of the diploid genome size (Table S13).

### Identifying the candidate DNMs

For each parent-offspring trio, the variants in three VCF files of each trio were filtered to a subset of single nucleotide variants (SNVs) by BCFtools (v1.21) (Danecek et al. 2021) and freebayes (v1.3.10) (Garrison and Marth 2012).

Variants were retained only if they met all of the following criteria: (i) the variant was located within the predefined callable genome regions; (ii) the variant had homozygous alternative allele status in the offspring's alignment against the contig with read depth > 10, and read depth of the inherited parent at the variant site of the contig was required to be >5; (iii) there were at most two alternative alleles at the variant site in each parental BAM file; (iv) the variant had a minimum QUAL score of 220, ensuring high-confidence base calls (BCFtools only); (v) no proximity to INDELs (outside ±20 bp of INDELs); (vi) variants not at the sites experiencing putative gene conversions described in a companion study (Tao et al. 2025), as gene conversions lead to read alignments resembling DNMs; and (vii) for a candidate DNM detected on a specific parental contig in an offspring, all other offspring did not contain a heterozygous genotype at the same site of the contig, as heterozygosity implies an inherited variant or alignment errors.

Regarding our treatment of gene conversion in the DNM pipeline, we note that distinguishing a true de novo point mutation from a non-crossover gene conversion event at heterozygous sites in a specific parent is challenging when the resulting nucleotide—whether due to mutation or conversion—is identical to the other nucleotide of the heterozygous site (e.g. a T>G mutation at a T/G site in a parent). However, in our companion recombination study (Tao et al. 2025), we showed that the probability of gene conversions at a heterozygous site in a parent is orders of magnitude higher than that of DNMs. Given this information, ambiguous events at these heterozygous sites are far more likely to represent gene conversions than DNMs. For this reason, and to avoid counting recombination-associated conversion tracts as point mutations, we conservatively excluded candidate sites showing gene-conversion–like patterns from the DNM set.

After applying these filters, the resulting set of candidate DNMs was further evaluated by importing both parental and offspring BAM files into IGV (v2.19.2) (Thorvaldsdottir et al. 2013) and using IGVtools to generate screenshots for each candidate DNM in both the offspring and the parents. Subsequent manual inspection led to the removal of any candidate DNMs if they met one or more of the following criteria: (i) structural variant/recombination breakpoint neighborhoods and (ii) variants were found to arise from misassigned parental origin of contigs, upon manual review (Figures S10 and S11). These steps yielded a DNM dataset for each individual (Table S6). The genome-wide mutation rate was calculated by the number of all DNMs divided by the sum of callable genome sizes of all offspring. The callable genome size was not multiplied by two, since the reconstructed inherited genomic segments effectively represent an approximately diploid genome for each offspring.

Because the coordinates of DNMs were located on the parental genomic contigs, the parental origin of the contig harboring each DNM naturally revealed its source, allowing for unequivocal determination of the DNM's parental origin without any further processing. This same principle applied to the callable genome as well.

### Simulation for assessing alignment errors if using the public reference genome

To assess alignment errors due to low sequence similarity between our sequencing data and the public *B. floridae* reference genome, we performed a simulation analysis. Artificial genomes were generated by introducing varying proportions of substitutions into the published *B. floridae* reference. Paired-end 150 bp reads were then simulated from these artificial genomes using ART (Huang et al. 2012) with parameters “-p -m 450 -s 50 -f 10 -l 150 -ef -ss HS25.” The simulated reads were aligned to the original *B. floridae* reference using BWA-MEM2 (v2.2.1) (Vasimuddin et al. 2019), and mis-mapped reads were quantified with a custom script. The results are summarized in Table S2.

### Simulation for assessing the FNR of DNM detection

For evaluating the FNR in our DNM detection pipeline, we generated simulated data based on callable genome regions of three selected offspring. For each of the three individuals, we made an artificial genome based on the callable genome regions of that individual and put a specific number of simulated DNMs within the artificial genome. We next generated 80× simulated paired-end 150 bp reads for each selected individual using ART (Huang et al. 2012) and re-ran the full alignment and DNM detection workflow. The detected DNMs were then compared with the known positions of simulated DNMs to estimate the FNR (the equation below) for each selected offspring independently. The final FNR value was reported as the average across the three simulations (details given in Table S14).


(1) $$FNR=1-\frac{n_{Detected\;DNM}}{n_{Simulated\;DNM}}$$

### DNM rate estimation

The per-site-per-generation mutation rate (µ) was calculated as


(2) $$\mu =\frac{n_{DNM}}{Length_{Callable\;}\times (1-FNR)}$$

*n_DNM _* is the number of all detected DNMs. The callable genome size was independently determined for each offspring using the procedures described above and the callable genome sizes were then summed across all offspring to obtain the total callable genome size (LengthCallable). The FNR of the DNM detection pipeline was estimated from the simulation described above. All candidate DNMs were visually reviewed in IGV (v2.19.2) (Thorvaldsdottir et al. 2013), and a subset was further validated by Amplicon-seq. These quality control steps ensured high confidence in DNM identification. As all putative false-positive DNMs were removed, we did not apply false discovery rate correction for mutation rate estimates.

### Analysis of nucleotide diversity in amphioxus populations

To estimate nucleotide diversity in *B. floridae* populations, we re-analyzed previously published population data. First, we downloaded WGS data of 35 *B. floridae* individuals (Huang et al. 2023) from NCBI's SRA database. The short-read alignment and variant calling largely followed the method of Huang et al. (2023), with key adaptations. Specifically, the *B. floridae* genome assembly of that study (Huang et al. 2023) was utilized as the reference, with reads mapped using BWA-MEM2 (v2.2.1) (Vasimuddin et al. 2019) under default parameters optimized for vertebrate genomes. We then generated gVCF files for each individual using GATK4 (v4.6.1.0) (Van der Auwera and O’Connor 2020) HaplotypeCaller, setting the “-ERC BP_RESOLUTION” to include non-variant sites in the output, and adjusting parameters to reflect the heterozygosity of amphioxus by parameters “–heterozygosity to 0.03 and –indel-heterozygosity to 0.003.” The sequence alignment and variant detection workflows were integrated into an automated nf-core/sarek pipeline (v3.4.0) (Garcia et al. 2020) with key parameters fine-tuned, thereby maximizing the utilization of parallel computing resources.

For variant sites, we performed initial filtering using GATK4 VariantFiltration on SNPs and INDELs independently, excluding SNPs with QUAL < 30, QD < 2, FS > 60, MQ < 40, MQRankSum < -12.5, Read PosRankSum < -8, or SOR > 3 and excluding INDELs with QUAL < 30, QD < 2, FS > 200, ReadPosRankSum < -20, and SOR > 10 (Mirchandani et al. 2024). For invariant sites, we filtered based on quality (“QUAL > 30”) and the fraction of missing genotypes at a site (“F_MISSING < 0.25”).

To estimate nucleotide diversity at synonymous sites (*π*_S_), we used the script dNdSpiNpiS (v1.0) (https://kimura.univ-montp2.fr/PopPhyl/index.php?section=tools) with the default parameters. To extract phase information for the CDS features, we used GenomeTools (gt gff3 -sort -tidy -retainids). Based on the VCF file, we then generated the diploid fasta sequence for each individual using the FastaAlternateReferenceMaker module from GATK (v4.6.1.0) (Van der Auwera and O’Connor 2020). We then intersected these fasta sequences with the gene annotation GFF file to keep only CDS with SNPs. For each CDS, individuals with missing data were excluded when estimating *π*_S_. As for *π*, these estimates were corrected to account for truly invariant sites versus those resulting from insufficient sequencing depth. We used the invariant sites generated above to estimate the expected percentage of bases called per CDS and applied this measure as a correction factor for the *π*_S_ estimates. The mean diversities *π*_S_ of the CDS were then calculated for the whole genome.

### Analysis of the effective population size

The average long-term *N*_e_ of *B. floridae* was estimated using the formula *N*_e_ = *π*/(4*μ*) (Kimura 1983), where *μ* was the mutation rate estimated by our work and *π*_S_ was estimated with data from a previous study (Huang et al. 2023) (Table S10). For comparison, *μ* and *π* data of vertebrates were from Bergeron et al. (2023).

Using the same pipeline as Bergeron et al. (2023), we applied PSMC (Li and Durbin 2011) to estimate the trajectories of changes in the effective population size. The resulting *N*_e_ trajectory was similar to that reported in a previous study of amphioxus (Bi et al. 2020). The harmonic mean *N*_e_ estimated from the PSMC analysis (Figure S4) is substantially lower than the *N*_e_ derived from the formula *π*/(4*μ*). We found that, due to the exceptionally high genomic heterozygosity, the variant-calling pipeline of Bergeron et al. (2023) identified only ∼2 million heterozygous variants—corresponding to an observed heterozygosity of ∼0.6%, far below the expected value. This discrepancy suggests that the absolute *N*_e_ values are probably underestimated. Consequently, for cross-species comparisons, we used the *N*_e_ for *B. floridae* derived from the formula *π*/(4*μ*).

### Aligning parental genome assemblies to the published *B. floridae* genome

Owing to inherent limitations of short-read sequencing, the assembly of parental genomes consisted of primarily short contigs. Defining orthologous relationships between contigs of two parents in a specific locus based solely on pairwise alignments of contigs, as previously mentioned, can be complicated by the variability in the lengths of assembled contigs derived from that locus. To address these issues, we aligned the contigs of parental genomes to a published *B. floridae* haploid reference genome (Huang et al. 2023). After testing multiple alignment tools under various parameter configurations, Minimap2 (v2.24) (Li 2021) was selected as the optimal aligner, with empirically optimized parameters: “-x asm20 –secondary = no.” Resultant alignments were processed through sequential refinement: (i) coordinate sorting via SAMtools (v1.21) (Li et al. 2009) and (ii) removal of multi-mapping regions using Sambamba (v0.8.2) (Tarasov et al. 2015). A custom script was employed to establish the mapping relationship between each parental genome and the haploid reference genome. This approach yielded a multiple genome alignment that aligned positions across the paternal, maternal, and reference genomes. As a result, the majority of parental genomic positions were successfully anchored onto the reference, thereby obtaining orthologous genomic coordinates across the parental genomes and facilitating subsequent analyses dependent on chromosomal coordinates.

### Comparative analysis of mutation rates in different contexts

To compare mutation rates in different contexts (e.g. different annotation groups), we utilized the coordinate mapping relationships described in the previous section. We mapped the gene functional annotations from the published *B. floridae* reference genome (Huang et al. 2023) onto the parental genomes. In doing so, the coordinates for exonic, intronic, and intergenic regions were converted to those of the parental genomes. This allowed us to determine the lengths of each gene feature where DNMs and callable genome regions were present, enabling the calculation of annotation-specific mutation rates for each individual.

To generate the mutation spectrum, the DNMs were also stratified into six groups according to their mutation types (A>C/T>G, A>T/T>A, A>G/T>C, C>A/G>T, C>G/G>C, and C>T/G>A), with a special group for C>T mutations at CpG sites. We extracted CpG positions from the parental genomes solely based on the CpG dinucleotide context, thereby allowing us to distinguish between CpG and non-CpG sites.

### Identifying the candidate PZMs

Raw VCF files of parent-offspring trios were obtained from the section “Alignment of offspring and parental reads to the parental reference genome and variant calling.” The filtering criteria for PZMs were largely analogous to those for DNMs, with three key differences: (i) we did not consider variants located in unphased regions as PZM candidates; (ii) unlike DNMs, heterozygous genotypes were retained; and (iii) we required that no other parental or offspring sample carried more than two alternative allele reads at the same genomic position. Generation of IGV screenshots and subsequent manual curation are similar to the procedures described in the section “Identifying the candidate DNMs” but with a few modifications, such as retaining heterozygous rather than strictly homozygous genotypes.

We next calculated the VAF of PZMs in the individual. First, we aligned all parental contigs to the published *B. floridae* reference (see “Aligning parental genome assemblies to the published *B. floridae* genome”) to establish orthologous relationships among allelic contigs. Using the inferred inheritance state in each offspring, we identified the allelic contig from the second parent and obtained read depths for both alleles at each PZM site via “bam-count.” These depths were then used to compute the VAF for each PZM in an offspring. Finally, we performed a statistical test (“prop.test” in R) to retain PZM candidates with VAFs that are not significantly greater than 0.25 (*P* > 0.05) because of their postzygotic origin.

### Amplicon-seq validation

To assess the reliability of DNMs across different data sources, we randomly selected a subset (Table S8) for validation by amplifying target regions with specific primers, followed by amplicon sequencing for genotyping. The validation workflow comprised three main steps: primer design, in silico PCR, and multiplex PCR amplification coupled with amplicon sequencing.

In the primer design phase, due to the high sequence polymorphism observed in *B. floridae* (∼4%), it was challenging to simultaneously amplify parental alleles using a single pair of primers targeting conserved regions; therefore, it became necessary to design allele-specific primers for each allele from the two parents. Specifically, we adopted the framework of the NGS-PrimerPlex (v1.3.4) (Kechin et al. 2020) multiplex PCR primer design process, with modifications tailored to our specific requirements. Since we needed to amplify all relevant alleles independently in offspring and parents, we chose the parental reference genome as the reference for primer design to maximize the number of alleles amplified by a single primer pair. For each locus, we used the mapping relationships between parental genomes, as described in the section “Aligning parental genome assemblies to the published *B. floridae* genome.” This allowed us to extract the target alleles harboring the DNMs and the orthologous alleles from the other parent. To avoid software misinterpretation of homologous regions as multiple alignments, we removed non-target contigs from the reference genome. We then used the processed parental reference genome for each individual together with the DNM positions to run NGS-PrimerPlex (parameters given in Table S7). After executing the pipeline, we selected the top five multiplex PCR primer sets with the highest primer scores as candidate primer combinations for the subsequent steps (Table S7).

In silico PCR was employed to confirm that primers designed for multiplex amplification can effectively amplify all parental alleles at the loci harboring selected DNMs, using *in_silico_pcr.pl* (v0.6, https://github.com/egonozer/in_silico_pcr). To optimize efficiency, only the contig harboring the DNM and orthologous contigs were extracted as the reference genome for in silico PCR. If in silico PCR showed incomplete allele amplification, we designed additional primers for the unamplified contig. These primers were added to the multiplex primer set, and the process was repeated until all target fragments could be amplified.

For multiplex PCR, we used the gDNA templates of offspring and parents stored previously and performed PCR amplification with a multiplex PCR kit (VAZYME, CN: PM101-02) optimized for both sensitivity and specificity; the resulting products are then subjected to agarose gel electrophoresis, and finally, DNA fragments ranging from 150 to 200 bp were excised from agarose gel following electrophoresis and subsequently purified using gel extraction kit. Library quality was assessed by fragment analysis using the Qsep-400 and quantified for concentration using the Qubit 3.0 Fluorometer (Thermo Fisher Scientific, Waltham, MA). Library construction was performed using the VAHTS Universal Plus DNA Library Prep Kit for Illumina ND617, following the manufacturer's protocol. Libraries meeting the quality control criteria were subsequently sequenced on the Illumina NovaSeq 6000 platform.

## Supplementary Material

msag017_Supplementary_Data

## Data Availability

The raw sequence data reported in this paper have been deposited in the Genome Sequence Archive (GSA: CRA027444) that are publicly accessible at https://ngdc.cncb.ac.cn/gsa. The raw sequence data of previously sequenced populations can be accessed under the BioProject with accession number: PRJNA602496. Genome assemblies produced by Platanus-allee and the amplicon sequencing data have been deposited in Science Data Bank (ScienceDB) repository (https://doi.org/10.57760/sciencedb.27648). Custom scripts used are available online at https://github.com/xuejinghub/Amphioxus_denovo_mutation_rate.
