# Supplementary material for: Germline de novo mutation rate of the highly heterozygous amphioxus genome
Source: Mol Biol Evol. 2026 Jan 14;43(2):msag017. doi: 10.1093/molbev/msag017 (PMC12862219; doi:10.1093/molbev/msag017)
Supplement: msag017_Supplementary_Data [file msag017_supplementary_data.zip › mutation rate Supplementary figures_1207.pdf]

Supplementary tables are provided in a separate Excel file, and supplementary figures are shown below.

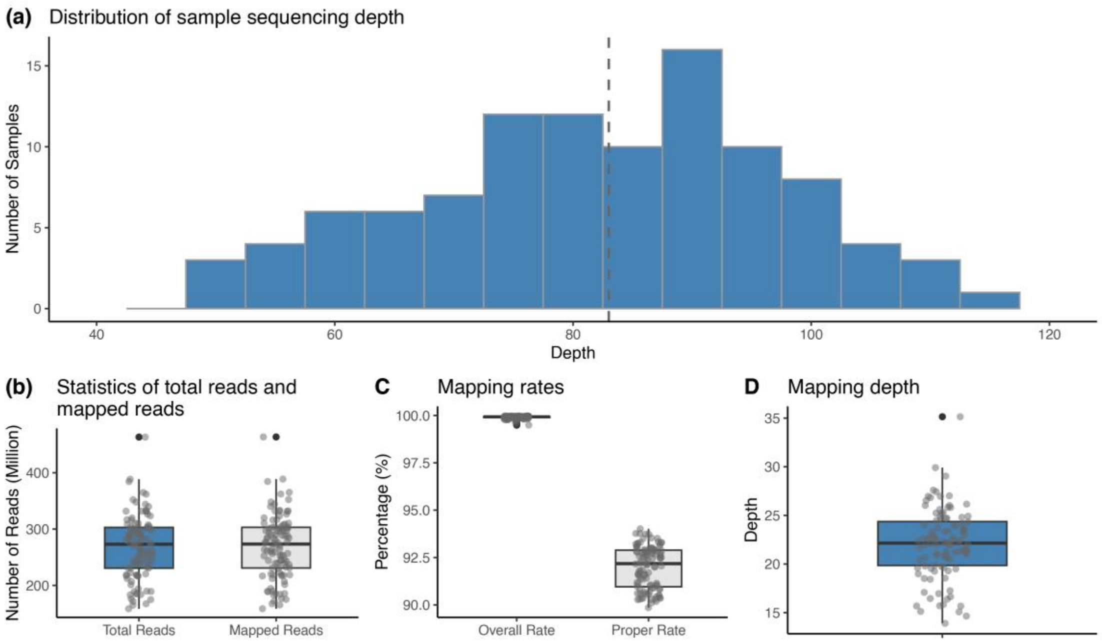

Figure. S1 Statistics of sequencing and mapping results. a) Distribution of sequencing depths of all samples. b) Boxplots showing the numbers of all reads and the numbers of mapped reads against the parental genomes. c) Boxplots showing the percentages of all reads that were mapped to the parental genome, and the percentage of properly aligned reads. 'Properly aligned' means that paired-end reads were aligned to the same sequence with an insert size of ≤ 1,000 bp. d) Boxplot showing average read depths of samples based on the alignments against the parental genome.

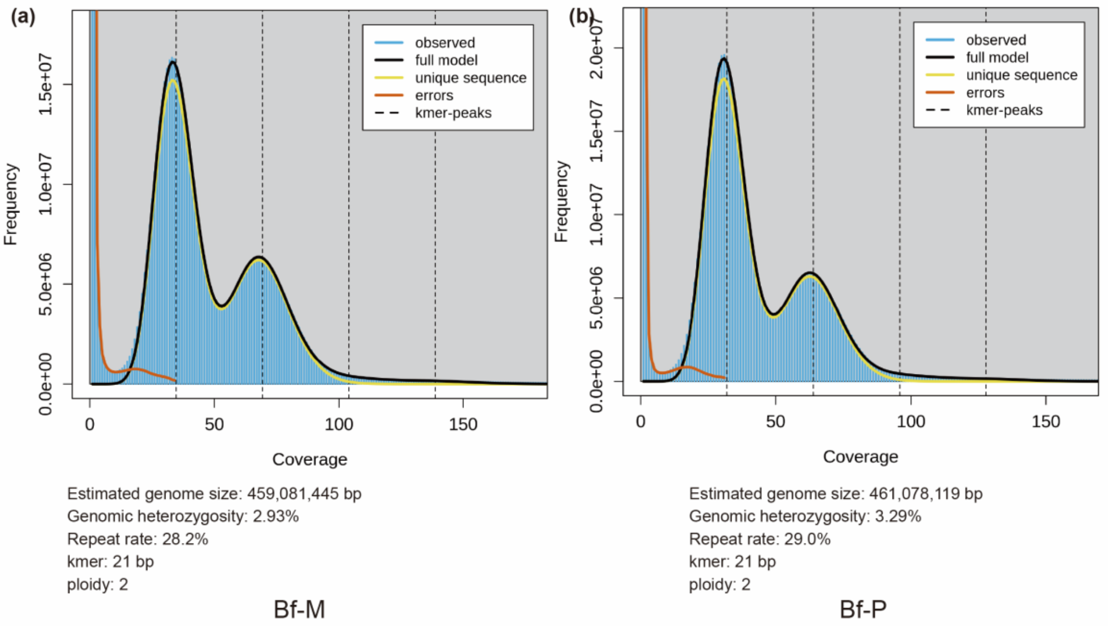

Figure. S2. Genome characteristics of *B. floridae* parental genomes based on 21-mer analysis. a) Maternal genome. b) Paternal genome. The bottom of each panel displays genome characteristics inferred from 21-mer analysis, including estimated genome size, genomic heterozygosity, repeat rate, *k*-mer size and ploidy.

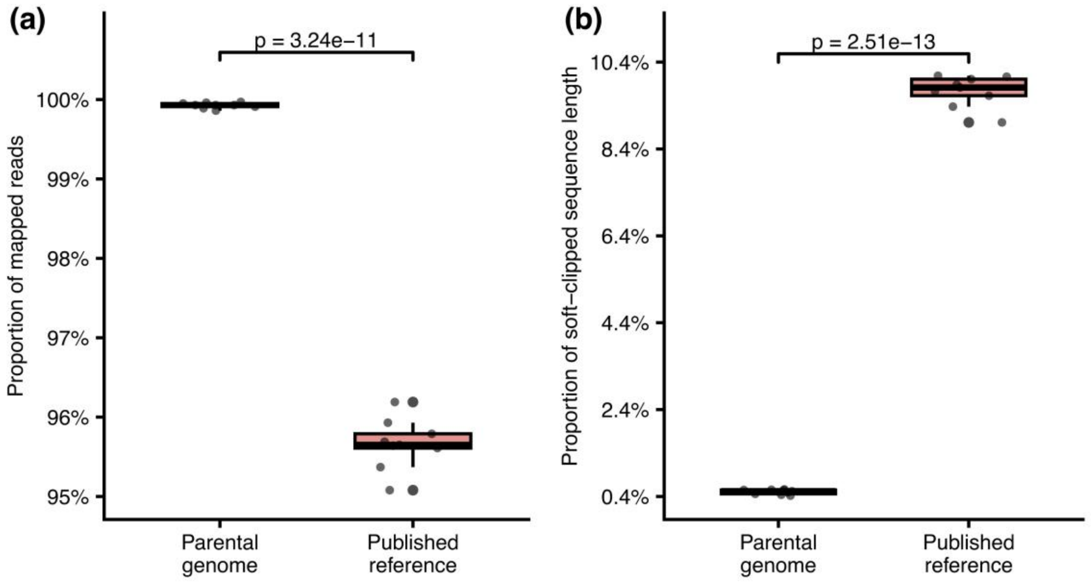

Figure. S3. Comparation of mapping rates (a) and the proportions of soft-clipped sequence lengths b) when short reads are aligned against different reference genomes. Reads of ten offspring were used to do this comparative analysis. Statistical significance was assessed using paired *t*-tests, and *P* values are indicated in the figure.

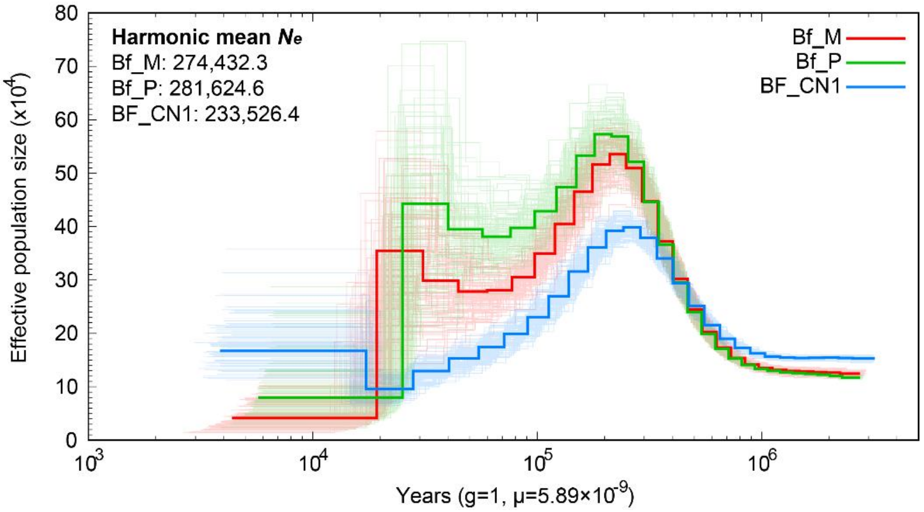

Figure. S4. Temporal dynamics of effective population size for three *B. floridae* individuals. Bf_M and Bf_P are two inbred samples from our study, and BF_CN1 is a laboratory-raised sample from Bi et al. (2020). The generation time (g) and the mutation rate (μ) were assumed to be 1 year and 5.89×10^{-9}, respectively. And the harmonic mean N_e was calculated based on the PSMC analysis for the period from 30,000 to 1,000,000 years ago. The bold curves show the estimates based on original data, and the light curves show the estimates for 100 bootstrapped sequences.

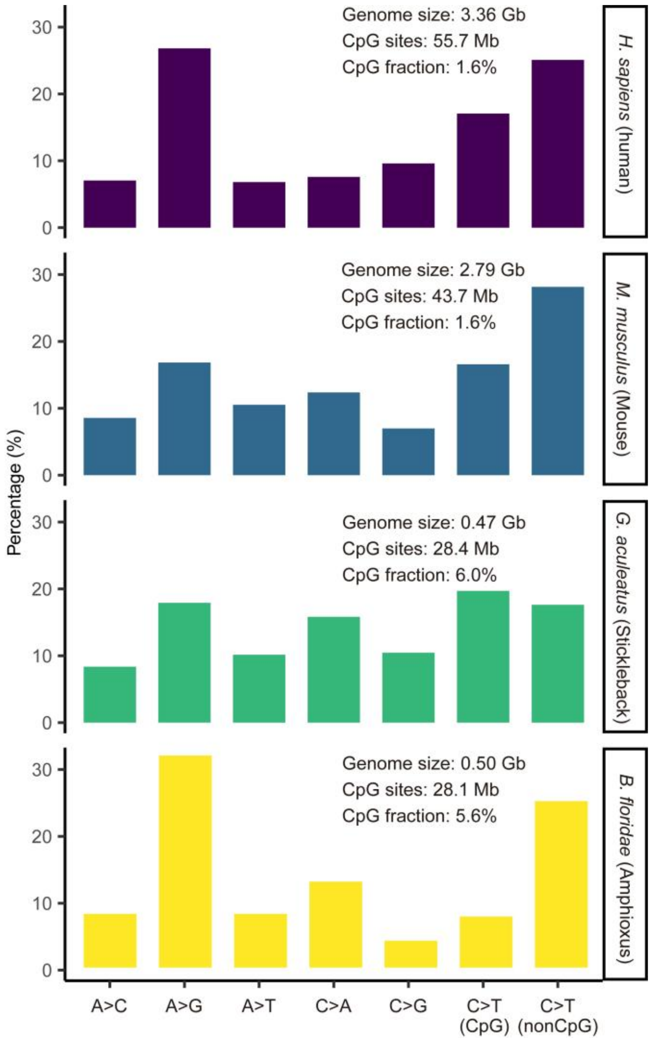

Figure. S5 Mutation spectra of *B. floridae* and representative vertebrate species. *H. sapiens* (Human), purple; *M. musculus* (Mouse), blue; *G. aculeatus* (Stickleback), green; *B. floridae* (Amphioxus), yellow.

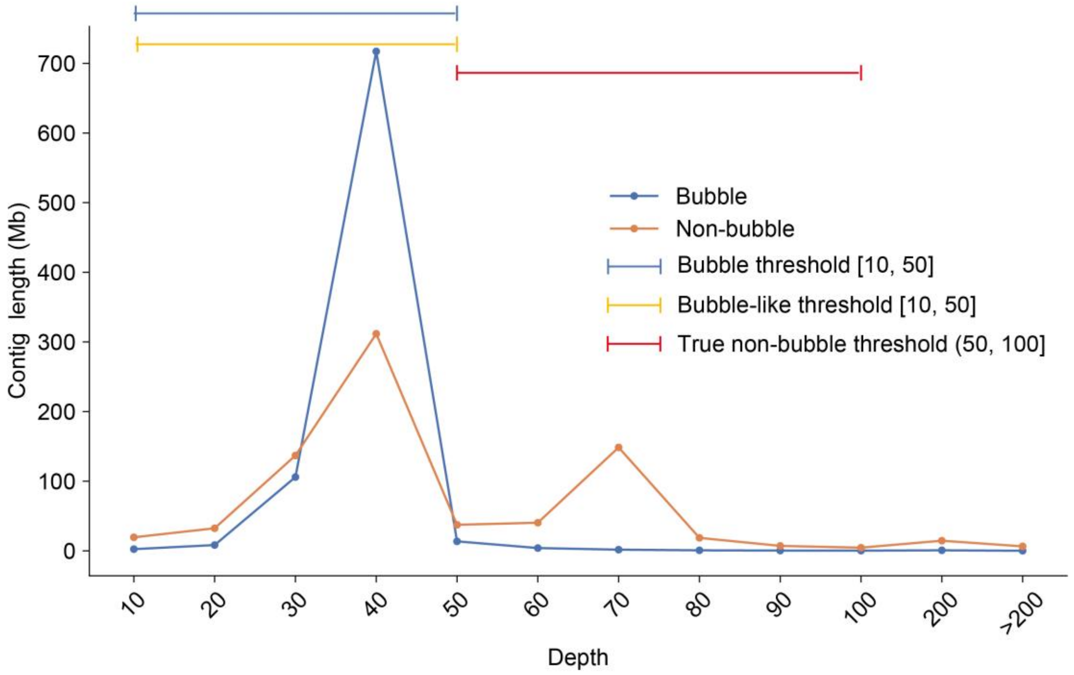

Figure S6 The distribution of mean read depths of bubble and non-bubble contigs (in terms of total contig length) in the parental assemblies. The thresholds used for partitioning bubble-like contigs and true non-bubble contigs in non-bubble contigs are shown in the figure.

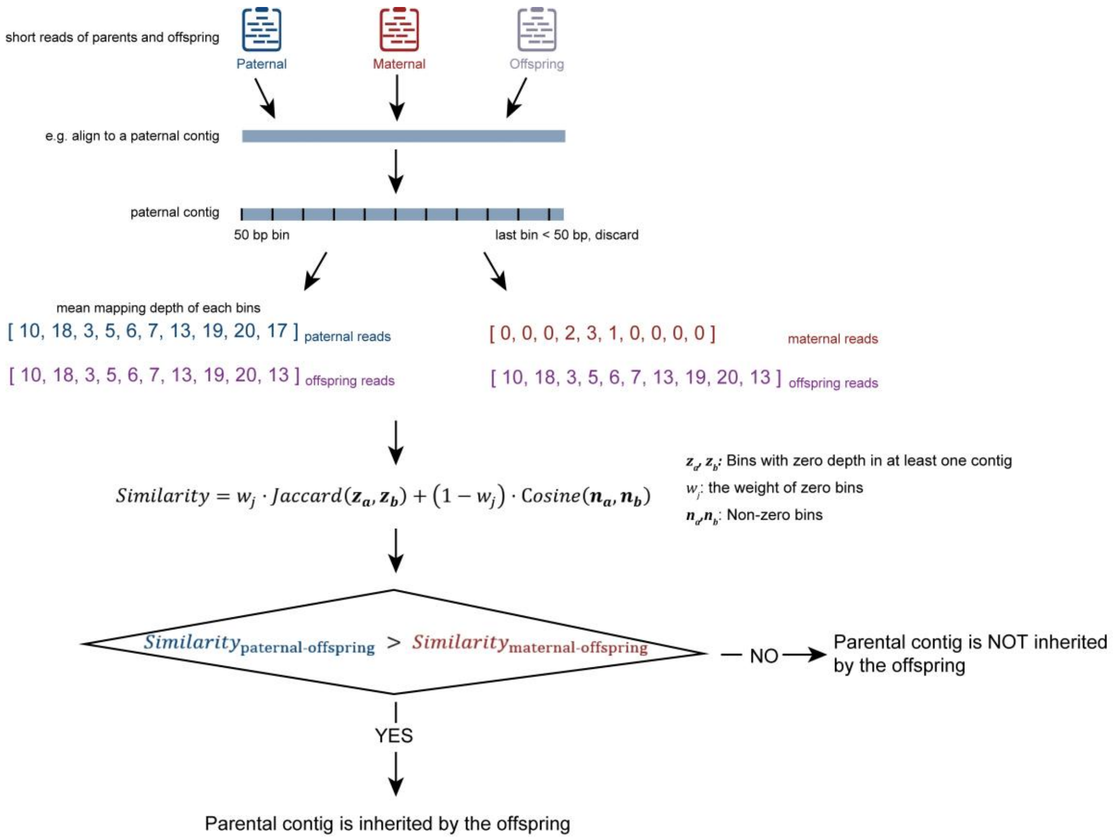

Figure. S7 Inferring inheritance states of parental contigs in offspring. First, we aligned parents' and offsprings' WGS reads to the parental reference genome. In the figure, we chose a paternal contig as an example. To quantify depth distribution, the paternal contig was divided into 50-bp bins, discarding any terminal bin shorter than 50 bp. The mean depth within each bin was calculated for the offspring, the mother and the father, respectively, generating a depth distribution vector per individual across the paternal contig. The similarity between offspring and parental depth vectors was quantified using a weighted measure combining: (1) Cosine similarity for bins with non-zero depths in both individuals being compared, and (2) Jaccard similarity for bins with zero coverage in at least one of the two individuals (accounting for regions with no aligned reads). The paternal contig was considered inherited by an offspring if the offspring's depth distribution vector showed greater similarity to that of the father than to the mother.

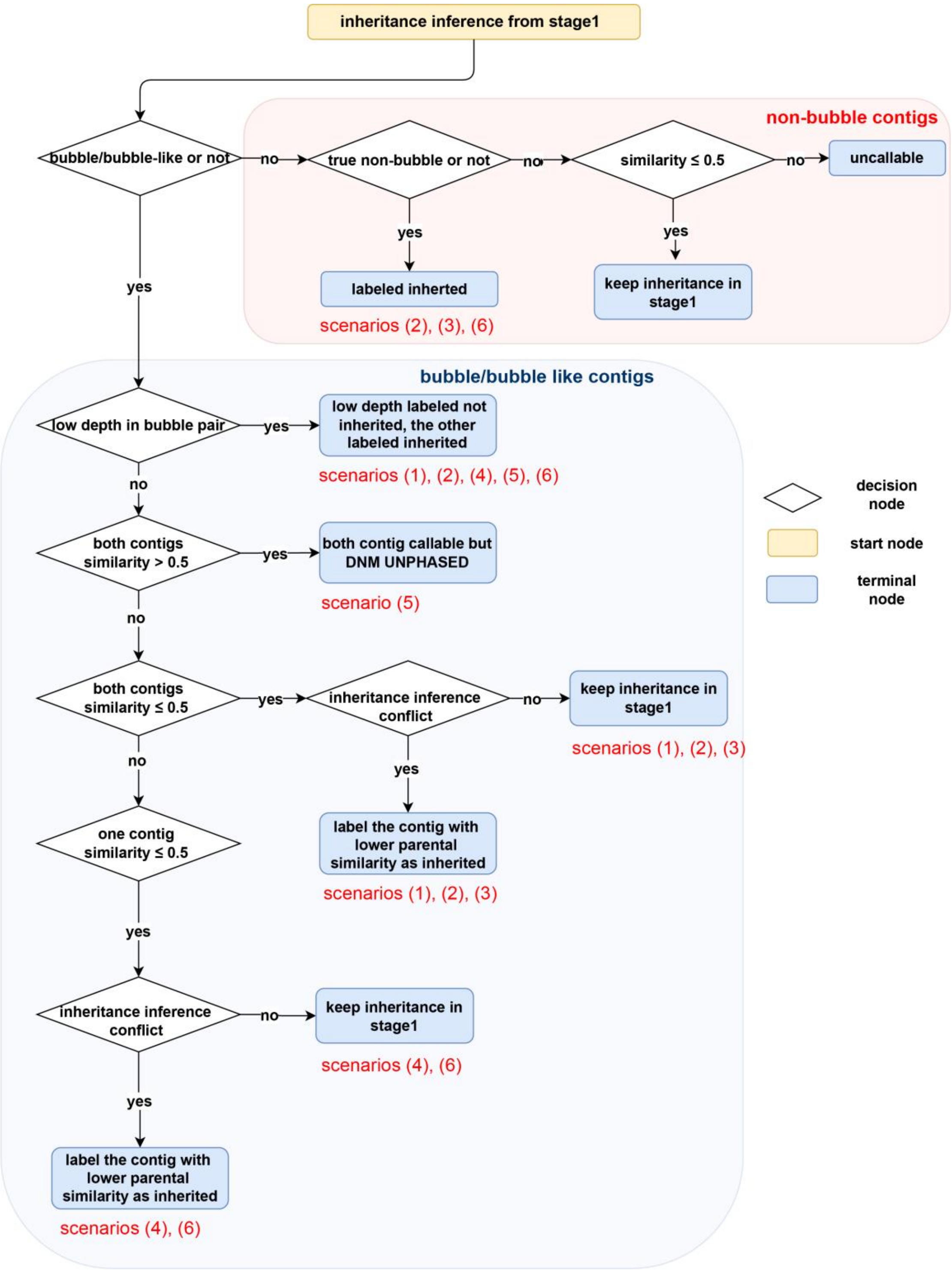

Figure S8 Flowchart for the stage 2 of inheritance inference. Yellow rectangles denote start nodes, corresponding to the stage 1 inheritance inference for each contig. Diamonds indicate decision nodes, and blue rectangles represent terminal nodes. The 'similarity' in the figure means the contig similarity between two alleles from two different parents. The red labels below terminal nodes indicate the corresponding scenarios illustrated in **Figure. S9**.

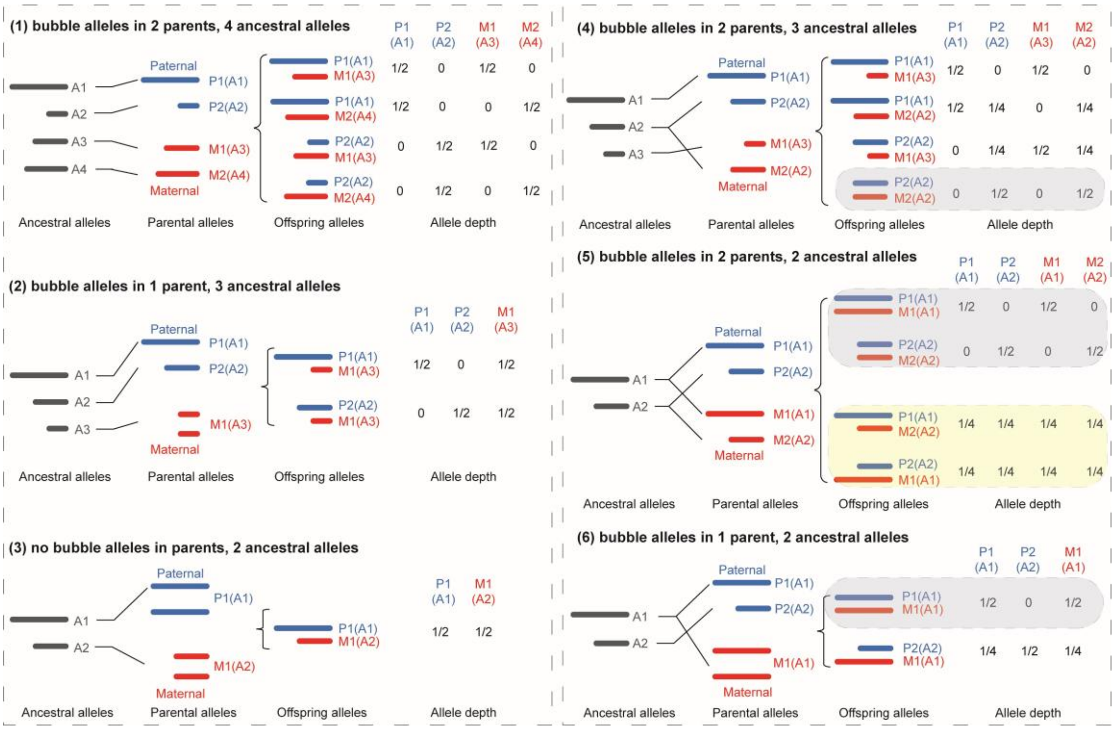

Figure S9 Possible allele combinations in the paternal and maternal genomes at a locus and possible resulting read depths of alleles in the offspring. Distinct alleles (assembled contigs) are named A1, A2, etc. P1, P2, M1 and M2 are names of paternal and maternal alleles. The value of an allele depth represents the relative ratio of read depth of the specific allele to an offspring's average read depth. For scenarios (1) - (3), the two parents do not have shared (highly similar) alleles, the inheritance states of alleles can be determined and the offspring alleles can be phased. In contrast, for scenarios (4) - (6), when two highly similar parental contigs are inherited together by an offspring, the inheritance state of the contigs themselves can still be inferred, but DNMs located on these contigs cannot be reliably detected, because PZMs can produce read mapping patterns similar to those of DNMs. Such regions are therefore excluded from the callable genome (gray shade). For the scenario (5) where a pair of bubble contigs in one parent are both similar to their allelic counterparts in the other parent, two outcomes are possible: a) if the similar contigs from the two parents are inherited, the situation is like the cases with gray shade in scenarios (4) and (6); b) if the dissimilar contigs are inherited (yellow shade), the inheritance state of the contigs in the offspring is uncertain, but any DNM present on these contigs can still be detected; these DNMs are counted as unphased DNMs in our analysis.

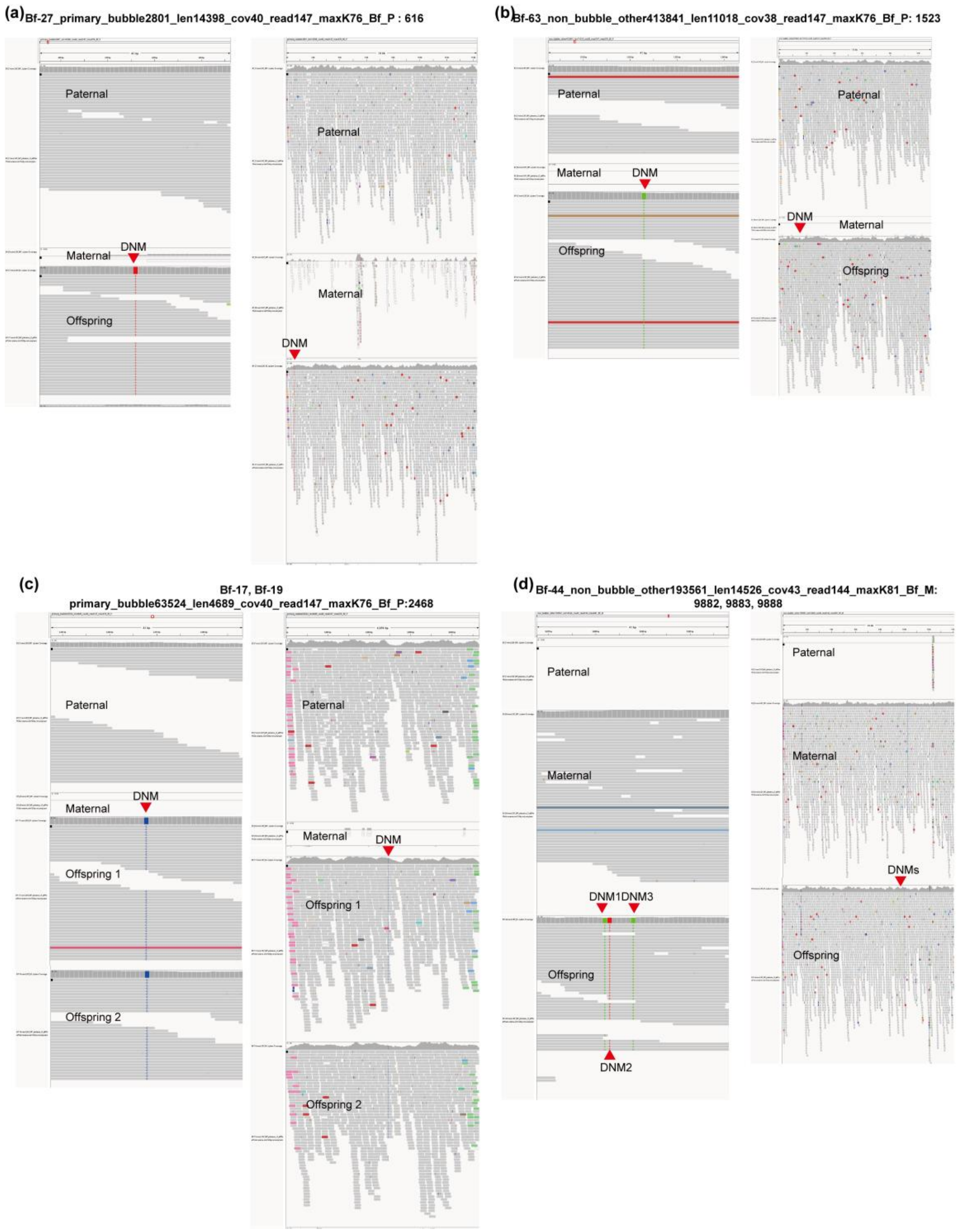

Figure S10 Example IGV snapshots of included DNMs. a) and b) are examples for single DNMs, c) for ssDNM, and d) for clustered DNMs. For each panel, the left screenshot shows a zoomed-in view spanning 20 bp upstream and downstream of the DNM to visualize individual base calls, whereas the right screenshot shows the entire contig to illustrate overall coverage. “Paternal”, “Maternal”, and “Offspring” indicate read alignments from each respective individual to the focal contig.

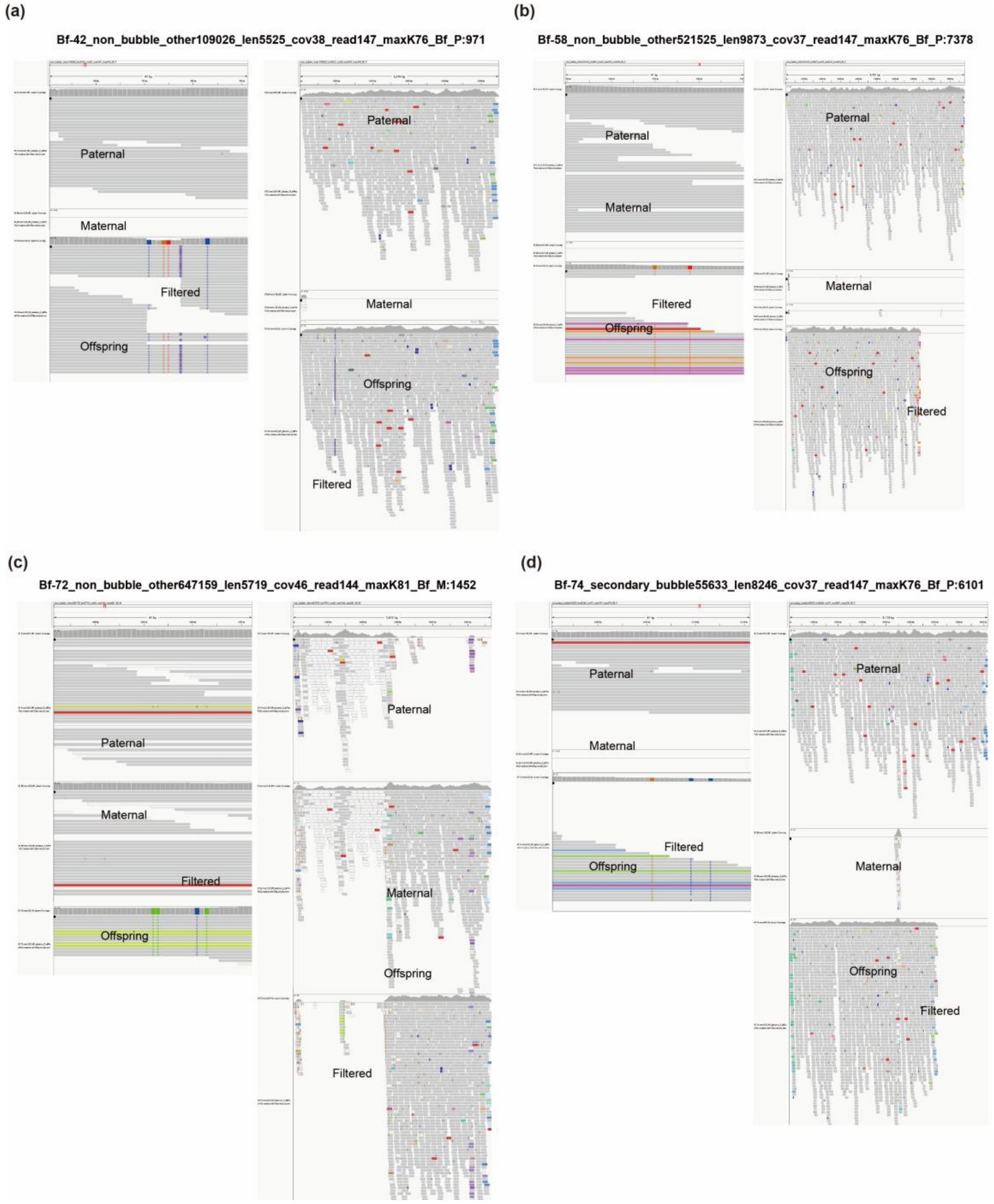

Figure S11 Example IGV snapshots of filtered DNM candidates. a) A DNM candidate located close to an indel that was missed by the automatic filtering. b) A candidate located at the breakpoint of a structural variant (SV) or recombination, because no offspring read mapped to the 3’ end of the inherited paternal contig. c) An example in which the inheritance pattern is formally consistent with transmission to the offspring, but different parts of the region likely contain an SV or recombination event. d) Another example of a potential SV/recombination breakpoint. In each panel, the left screenshot shows a zoomed-in view spanning 20 bp upstream and downstream of the

candidate variants to visualize individual base calls, whereas the right screenshot shows the entire contig to illustrate overall coverage. “Paternal”, “Maternal” and “Offspring” denote the alignments of reads from each individual to the focal contig, and “filtered” marks the position of a DNM candidate that was removed by our filters.
